# Trained immunity in monocyte/macrophage: Novel mechanism of phytochemicals in the treatment of atherosclerotic cardiovascular disease

**DOI:** 10.3389/fphar.2023.1109576

**Published:** 2023-02-21

**Authors:** Jie Wang, Yong-Mei Liu, Jun Hu, Cong Chen

**Affiliations:** Guang’anmen Hospital, China Academy of Chinese Medicine Sciences, Beijing, China

**Keywords:** trained immunity, monocyte/macrophage, atherosclerosis, natural products, epigenetic reprogramming, metabolic reprogramming

## Abstract

Atherosclerosis (AS) is the pathology of atherosclerotic cardiovascular diseases (ASCVD), characterized by persistent chronic inflammation in the vessel wall, in which monocytes/macrophages play a key role. It has been reported that innate immune system cells can assume a persistent proinflammatory state after short stimulation with endogenous atherogenic stimuli. The pathogenesis of AS can be influenced by this persistent hyperactivation of the innate immune system, which is termed trained immunity. Trained immunity has also been implicated as a key pathological mechanism, leading to persistent chronic inflammation in AS. Trained immunity is mediated *via* epigenetic and metabolic reprogramming and occurs in mature innate immune cells and their bone marrow progenitors. Natural products are promising candidates for novel pharmacological agents that can be used to prevent or treat cardiovascular diseases (CVD). A variety of natural products and agents exhibiting antiatherosclerotic abilities have been reported to potentially interfere with the pharmacological targets of trained immunity. This review describes in as much detail as possible the mechanisms involved in trained immunity and how phytochemicals of this process inhibit AS by affecting trained monocytes/macrophages.

## 1 Introduction

Atherosclerotic cardiovascular diseases (ASCVD) have emerged as the most common burden of disease as a result of the aging and expanding global population (Mensah et al., 2019). As the pathology of ASCVD, atherosclerosis (AS) generates a continuous buildup of vessel-occluding plaques in the subendothelial intimal layer of coronary arteries, eventually leading to considerable blood flow restriction and essential tissue hypoxia (Libby, 2002; Gallino et al., 2014). Most cardiovascular events are caused by the rupture of atherosclerotic plaques in the arterial artery wall and the subsequent formation of an occluding thrombus.

In addition to the deposition and retention of modified lipoproteins and the buildup of immune cells in the walls of major arteries, AS is characterized by a low-grade, persistent, chronic inflammation of the arterial wall (Edgar et al., 2021). All phases of AS are mostly attributed to monocytes and monocyte-derived macrophages, which are also thought to be responsible for persistent chronic inflammation (Moore et al., 2013). The traditional view is that innate immune cells, such as macrophages, can only eliminate pathogens non-specifically through biological processes such as phagocytes (Bonilla and Oettgen, 2010). However, a growing body of research suggests that monocytes/macrophages may also develop memory capabilities similar to those of the adaptive immune system after exposure to pathogens (Arts et al., 2018). Myeloid cells of the innate immune system become more sensitive after activation with the same or different stimuli to produce a persistent inflammatory monocyte/macrophage phenotype, a phenomenon known as “trained immunity” or “innate immunological memory” (Netea et al., 2020). This persistent overactivation of the innate immune system could contribute to the incessant vascular wall inflammation that is characteristic of AS (Moore et al., 2013).

For thousands of years, herbal medicines have been widely utilized alone or as a supplementary strategy to treat various disorders in East Asia because of their reduced toxicity, fewer side effects, and cheaper cost (Wang et al., 2018). Along with the development of these natural therapies, herbal medicine is becoming more widely accepted as a supplement and alternative therapy in many countries (Liang et al., 2021). According to the most recent statistics on US-FDA (United States Food and Drug Administration) authorized drugs, herbal remedies have been a vital source of novel medications (Newman and Cragg, 2020). A growing body of scientific evidence has revealed that natural medicines and phytochemicals from natural herbal medicines exhibit promising anti-AS properties (Zhang et al., 2021a). Based on the regulation of targeting trained immunity in monocyte and macrophage, natural compounds generated from herbal remedies are surely excellent resources for selecting potential therapeutics to treat AS.

This review aims to provide more information on the role of trained immunity in the pathophysiology of AS, which might be a potential pharmacological target of natural products.

## 2 Trained immunity in AS

Conventional wisdom generally considers the adaptive immune system as a specific protective mechanism that can form more specialized lines of defense against re-infection with the same pathogens (Domínguez-Andrés et al., 2019). However, innate immune cells (e.g., macrophages/monocytes) have been reported to display similar immune memory, referred to as trained immunity (Conrath et al., 2015; Milutinović and Kurtz, 2016; Gourbal et al., 2018). Studies of gene-specific chromatin changes brought about by lipopolysaccharide (LPS) have first shown trained immunity characteristics of monocytes/macrophages (Foster et al., 2007). Subsequently, infectious stimuli, such as β-glucan and Bacille Calmette–Guérin (BCG), improved their reactivity to stimulation with unrelated infections or molecular patterns linked with those pathogens (Quintin et al., 2012; Saeed et al., 2014). Factors that contribute to the development of AS, such as uric acid and oxidized low-density lipoprotein (oxLDL), and other endogenous ligands can activate trained immunity (Bekkering et al., 2014; Crişan et al., 2017). Freshly isolated monocytes from patients who had symptoms of coronary artery disease (CAD) had a higher capacity to produce cytokines than those from healthy controls, and this capacity was maintained following *ex vivo* conversion to macrophages for 5 days (Shirai et al., 2016). The atherogenic factors are characterized by increased production of proatherogenic cytokines and chemokines like tumor necrosis factor-α (TNF-α), IL-6, monocyte chemoattractant protein-1 (MCP-1), matrix metalloproteinases 2 (MMP-2), and MMP-9 and increased foam cell formation is indeed demonstrated by large-scale phenotyping of trained macrophages *in vitro* (Bekkering et al., 2014).

**FIGURE 1:**
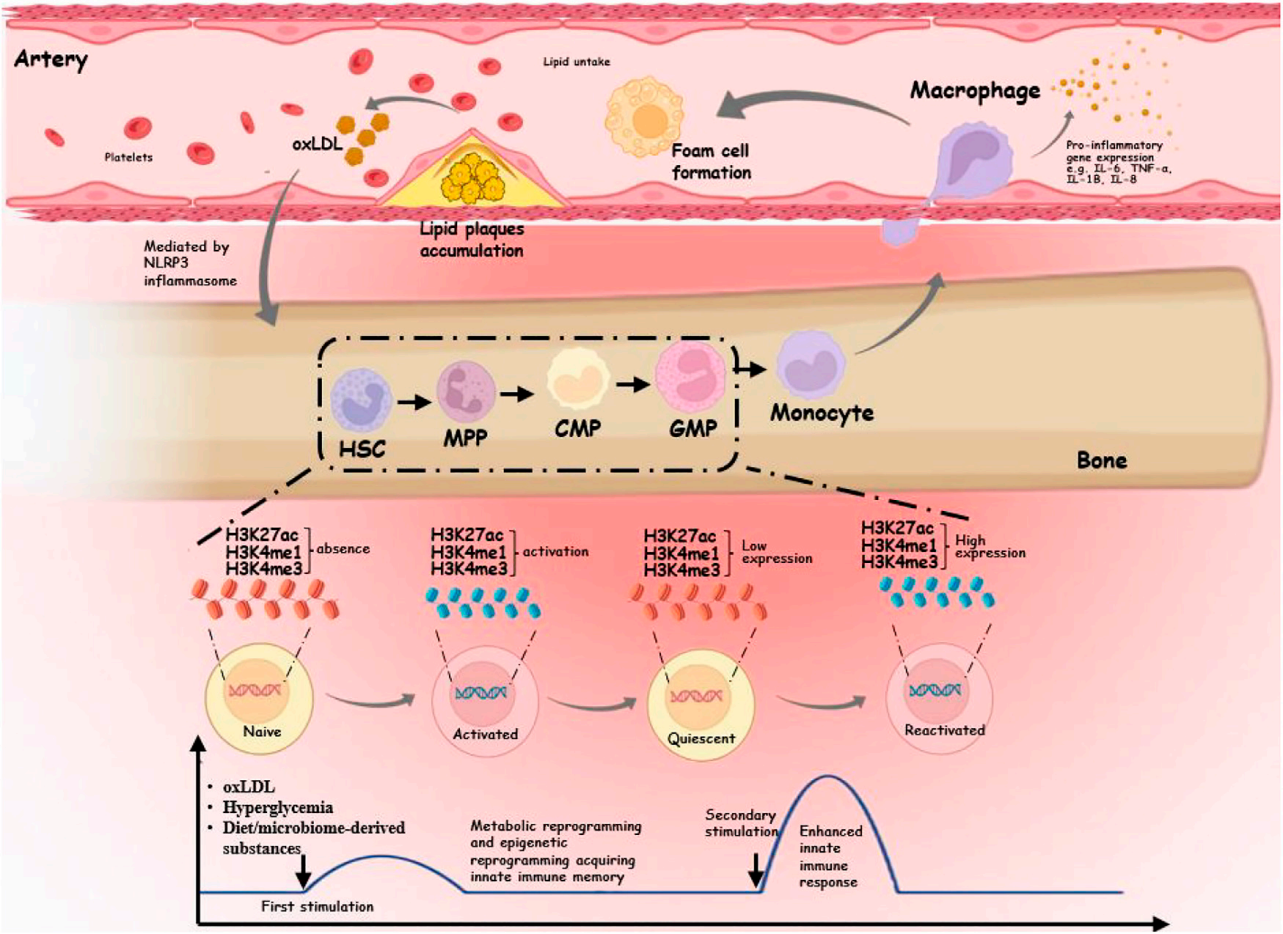
Schematic diagram of the trained immunity mechanism in atherosclerotic cardiovascular disease. In the hematopoietic system, myeloid cells exposed to endogenous triggers undergo epigenetic and metabolic reprogramming, resulting in acquiring innate immune memory. The initial gene activation is accompanied by the accumulation of H3K4me3 on the gene promoter, and the persistence of H3K4me1 or H3K27ac in secondary stimulation leads to an enhanced innate immune response. These trained myeloid cells differentiate into monocytes, which travel further into the intima to become macrophages. Trained macrophages produce high levels of proinflammatory cytokines such as TNF-α, IL-6, IL-8, and IL-18 and uptake of lipids to form foam cells. When plaques form, endogenous stimuli may be further released to form trained immunity mediated by the NLRP3 inflammasome.

After short activation with endogenous ligands, a persistent proinflammatory phenotype can emerge in AS monocytes/macrophages (Leentjens et al., 2018). The three key components of trained immunity are metabolic reprogramming, epigenetic reprogramming, and the promotion of myelopoiesis progenitors (Fanucchi et al., 2021) (Figure 1). First, metabolic reprogramming is responsible for the induction, maintenance, and regulation of trained immunity. Different metabolic pathways supply the required substrates for altering the structure of the respective sections of the chromatin and genome, in addition to acting as a source of energy and building components for the dynamic remodeling of the epigenetic landscape. Second, epigenetic reprogramming ultimately links metabolic changes to a cell’s gene expression and inflammatory phenotype. Finally, trained immunity in bone marrow by hematopoietic stem cells (HSCs) maintains long-term effects on circulating monocytes through differentiation into progenitor and mature cells (Mitroulis et al., 2018). In addition to atherosclerotic triggers, such as lipoproteins, glucose, diet, and microbiota-derived substances, proinflammatory cytokines secreted by monocytes/macrophages with an inflammatory phenotype may alter the tissue microenvironment by altering macrophages, the functional state of cells, leading to a vicious circle (Groh et al., 2018).

### 2.1 Metabolic reprogramming of trained immunity in AS

The trained immune activation has to quickly access a supply of substrates to initiate the numerous metabolic processes associated with the immune response. Intracellular metabolic pathways of glucose, amino acids, lipids, and nucleic acids are altered in response to trained immune activation (Fanucchi et al., 2021). When normal cells are at rest, they obtain enough energy *via* metabolic processes that are extremely effective but rather slow, such as oxidative phosphorylation (OXPHOS) and fatty acid oxidation (FAO) (Augert et al., 2020). In contrast, trained immune cells continue to opt for “aerobic glycolysis,” which uses glycolysis instead of OXPHOS to generate energy under normoxic conditions, similar to the “Warburg effect” in cancer (Mills et al., 2016; Renner et al., 2017). In addition to glucose metabolism, trained immune cells exhibit altered lipid and amino acid metabolic patterns. For instance, the Krebs cycle’s anabolic redefinition to synthesize cholesterol and phospholipids from citrate and acetyl coenzyme A (CoA) is a crucial metabolic event in trained monocytes (Arts et al., 2016a). When exposed to β-glucan, cholesterol synthesis is increased, but fluvastatin, an inhibitor of the enzyme 3-hydroxy-3-methylglutaryl-coenzyme A (HMG-CoA) reductase, inhibits trained immunity by downregulating histone H3 lysine 4 trimethylation (H3K4me3) and limiting the production of proinflammatory cytokines (Bekkering et al., 2018). For the progression of AS, the control of cholesterol import and efflux is critical. Similar to glutamine, arginine, and glycine, several particular amino acids are overexpressed in atherosclerotic plaques and have AS-promoting effects (Mallat et al., 1999; Sheehan et al., 2011). The intermediate metabolites from many metabolic pathways, such as aerobic glycolysis, glutaminolysis, cholesterol metabolism, and fatty acid synthesis, not only are a source of energy for the cell but also play several significant biological roles (Groh et al., 2018). Additionally, some chemo drugs made from natural herbal products, such as resveratrol and epigallocatechin gallate, can prevent cells from reprogramming their metabolism in response to AS.

#### 2.1.1 Glucose metabolism and AS

Although OXPHOS produces ATP more efficiently than other cellular processes (approximately 30 ATP molecules can be produced per glucose molecule during OXPHOS, whereas glycolysis can only produce two ATP molecules per glucose molecule) (Tabas and Bornfeldt, 2020). However, glycolysis produces ATP faster than OXPHOS, allowing immune cells to respond quickly to stimuli (Tabas and Bornfeldt, 2020). A clinical trial found an enhanced capacity for cytokine production in circulating monocytes obtained from ASCVD patients, which was associated with the upregulation of glycolytic enzymes (Bekkering et al., 2016; Shirai et al., 2016). This phenotype continued even after *in vitro* macrophage differentiation, displaying a greater glycolytic flux and a higher oxygen consumption rate (Shirai et al., 2016). Furthermore, the inhibition of specific tricarboxylic acid (TCA) cycle steps that support inflammatory processes is associated with increased glycolysis in inflammatory macrophages that primarily produce and release proinflammatory mediators, such as interleukin-1 (IL-1), TNF-α, chemokine C–C motif ligand 2 (CCL2), IL-12, and nitric oxide (NO), through inducible nitric oxide synthase (iNOS). The key proteins involved in glycolysis are introduced in the following sections.

##### 2.1.1.1 GLUT1

Glucose transporter 1 (GLUT1; gene name SLC2A1) on the cell membrane initiates glucose uptake by monocytes/macrophages (Fukuzumi et al., 1996). LPS and oxLDL, which cause inflammation, can boost GLUT1 expression and thereby increase glucose influx. Hexokinase phosphorylates glucose inside the cell to produce glucose-6-phosphate, which is then utilized in the pentose phosphate pathway (PPP), fatty acid synthase (FAS), or glycolysis. When glucose is processed in the cytosol by glycolysis, two ATPs and pyruvates are produced. Pyruvate produced during glycolysis either enters the mitochondrial TCA cycle or is transformed to lactate by lactate dehydrogenase (Christofk et al., 2008). In plaque macrophages, GLUT1 can promote antiatherosclerotic activities. The efferocytosis procedure increases the expression of GLUT1, which promotes an increase in glucose absorption and a transition from OXPHOS to improved aerobic glycolysis, both of which are required for the effective clearance of apoptotic cells (Morioka et al., 2018). When myeloid-targeted LysM-Cre Slc2a1^fl/fl^ animals were transplanted into Ldlr^−/−^ mice on a Western-style diet (WTD), the amount of necrotic core in the aorta increased (Morioka et al., 2018). Another study revealed that GLUT1 deletion in hematopoietic cells inhibited myelopoiesis, monocyte recruitment to lesions, and the progression of AS in ApoE^−/−^ mice, indicating that the main role of GLUT1 in this model was to encourage the proliferation of bone marrow HSCs and multi-potential progenitors, as well as the commitment of these cells to the bone marrow (Sarrazy et al., 2016).

##### 2.1.1.2 HIF-1α

Hyperoxia-inducible substance 1α (HIF-1α) is activated in hypoxic circumstances, allowing cells to switch to glycolysis and create ATP when oxygen is limited. Low oxygen levels trigger the HIF-1α transcription factor to initiate glycolytic metabolism, which decreases the need for OXPHOS and increases the expression of the key glycolysis proteins GLUT1, hexokinase II (HK-II), and 6-phosphofructo-2-kinase/fructo-2, 6-bisphosphatase (PFKFB3), which increases glycolytic flux (Tawakol et al., 2015). The activated macrophages will emit a lot of cytokines and absorb a lot of glucose. Indeed, hypoxia, HIF-1α expression, and FDG (fluorodeoxyglucose) uptake in macrophages are associated with atherosclerotic plaques in animal models of AS (Folco et al., 2011; Tawakol et al., 2015; Aarup et al., 2016).

#### 2.1.2 Lipid metabolism and AS

In homeostasis, lipoproteins taken up by macrophages are transported to lysosomes for the hydrolysis of cholesteryl esters. Free cholesterol is transported to the cytoplasm, where it is transported to the cell membrane for export or transported to the ER for re-esterification and storage in lipid droplets (LDs). Macrophages in advanced plaques provide signs of huge accumulations of free cholesterol, which suggests a breakdown in the mechanisms that keep cholesterol levels in balance. Membrane damage and metabolic dysregulation in the ER and mitochondria are required to maintain macrophage cholesterol homeostasis and reduce inflammation, which results from excessive accumulation of free cholesterol. Furthermore, high levels of modified cholesterol, oxLDL, are taken up by macrophages to form foam cells and promote plaque by secreting numerous proinflammatory cytokines and chemokines and producing MMPs that degrade plaque extracellular matrix pathogenesis (Khokha et al., 2013; Tall and Yvan-Charvet, 2015). It is reported that the induction of trained immunity in monocytes required stimulation of the cholesterol biosynthesis pathway but not cholesterol synthesis itself. oxLDL is an endogenous ligand that triggers trained immunity to activate monocytes/macrophages (Bekkering et al., 2014; Crişan et al., 2017). Another crucial characteristic of monocytes trained on β-glucan is increased cholesterol production (Netea et al., 2020). In primary human monocytes, fluvastatin, an inhibitor of HMG-CoA reductase, inhibits trained immunity (Arts et al., 2016a). Notably, β-glucan-induced training of mature myeloid cells and their progenitors requires enhanced cholesterol production. The accumulation of cholesterol esters and lipids with more saturated acyl chains is associated with the long-term myelopoiesis bias that β-glucan induced training imparts to HSCs (Mitroulis et al., 2018). The HSC population increase and myelopoiesis caused by β-glucan are reduced by HMG-CoA reductase inhibitor (Mitroulis et al., 2018).

#### 2.1.3 Amino acid metabolism and AS

Under AS pathological conditions, amino acid metabolites play an important role in supporting the induction, maintenance, and regulation mechanisms of trained immunity (Napoli et al., 2006). Therefore, it is necessary to decipher the role of specific amino acid metabolites in the induction of trained immunity.

##### 2.1.3.1 Glutamine

Glutamate is one of the amino acids that has been well-studied for its role in controlling inflammation (Wallace and Keast, 1992). By directly converting into glutamate, α-ketoglutarate, and succinate semialdehyde, glutamine contributes to the TCA cycle (Jha et al., 2015). Additionally, glutamate can be employed as a source of citrate for the FAS-catalyzed production of fatty acids. Recent research has demonstrated that glutaminolysis is increased in trained macrophages and is essential for the establishment of a trained macrophage phenotype in response to β-glucan (Arts et al., 2016a). In a different research study, oxidized phospholipids made of 1-palmitoyl-2-arachidonoyl-sn-glycero-3-phosphorylcholine (oxPAPC) were exposed to macrophages, which led to the development of AS, glutaminolysis, and IL-1 ^48^. This study demonstrated that in contrast to macrophages activated with LPS alone, those exposed to oxPAPC and LPS together had a metabolic change (Di Gioia et al., 2020). This metabolic shift was characterized by increased mitochondrial respiration, glutaminolysis, and accumulation of oxaloacetate, which stabilized HIF-1α and increased IL-1β production. IL-1β immunoreactivity in CD68^+^ lesional cells decreased in mice with systemic suppression of this pathway, which also reduced early AS (Di Gioia et al., 2020). Furthermore, the TCA cycle’s glutamine replenishment causes fumarate to accumulate, which integrates immunological and metabolic circuits to cause monocyte epigenetic reprogramming by inhibiting KDM5 activity and boosting the methylation of histone lysine 4 residues (Arts et al., 2016a). An epigenetic program identical to trained immunity-mediated by β-glucan was induced by fumarate. To support this, glutaminolysis inhibition and cholesterol production suppression in mice decreased the induction of trained immunity by β-glucan (Arts et al., 2016a).

##### 2.1.3.2 Arginine

In the context of AS pathology, arginine metabolism and its by-product NO are critical for the early stages of the disease (Lv et al., 2021). iNOS is ubiquitously expressed in activated and developing macrophages. NO creation by arginine is probably a factor in the metabolic transition. NO has been reported to inhibit OXPHOS in activated dendritic cells and inflammatory macrophages downstream of iNOS (Everts et al., 2012; Van den Bossche et al., 2016). Conversely, in alternatively activated macrophages and in the macrophages of atherosclerotic lesions that are regressing, Arg1 converts arginine to putrescine (Willecke et al., 2015). Under specific conditions, these two arginine metabolic routes can inhibit one another. As a result, NO inhibits ornithine decarboxylase’s ability to catalyze the conversion of ornithine to putrescine by S-nitrosylation a cysteine that is essential for the enzyme’s ability to function (Bauer et al., 2001). Conversely, ornithine decarboxylase prevents macrophages from becoming activated in an inflammatory response (Hardbower et al., 2017). Arginine metabolism has been reprogrammed, which promotes proinflammatory and healing processes.

##### 2.1.3.3 Serine

Recent research has demonstrated that LPS-activated macrophages promote serine synthesis, PPP, and one-carbon metabolism, which synergistically drive epigenetic reprogramming of IL-1β expression. The production of S-adenosylmethionine (SAM) during LPS-induced inflammation is fueled by the synergistic integration of glucose-derived ribose and one-carbon units supplied by glucose and serine metabolism into the methionine cycle through *de novo* ATP synthesis. Impairment of these metabolic pathways that feed SAM generation leads to anti-inflammatory outcomes (Yu et al., 2019). According to a different research study, serine is necessary for the synthesis of glutathione and IL-1β through the action of glycine (Rodriguez et al., 2019).

### 2.2 Epigenetic reprogramming of trained immunity in AS

Regulating gene expression without changing the DNA sequence itself is referred to as epigenetic reprogramming. Epigenetic reprogramming enables innate immune cells to react to future stimuli with a stronger, quicker, or qualitatively different transcriptional response (Zarzour et al., 2019). Epigenetic regulatory mechanisms encompass diverse molecular processes, including histone post-translational modifications, DNA methylation, and long non-coding RNAs (lncRNAs).

#### 2.2.1 Histone modifications

Epigenetic reprogramming occurs primarily through histone changes at the level of the chromatin structure to promote a sustained enhanced functional state of trained innate immune cells (Saeed et al., 2014). Neutralization of the positive charge of lysine residues in histones by histone acetylation increases the binding of transcription factors activating gene transcription (Bannister and Kouzarides, 2011). The specific lysine residue implicated and the sum of the additional methyl groups determine the effect of histone methylation on gene transcription. Two important epigenetic marks for trained immunity are as follows: the H3K4me3 accumulation at the gene promoter and the histone 3 lysine 27 acetylation (H3K27ac) acquisition at the gene’s distal enhancer (generated by histone 3 lysine acid 4 methylation (H3K4me1)) (Netea et al., 2020).

#### 2.2.2 DNA methylation

DNA methylation is involved in the regulation of patterns of gene expression. DNA methyltransferases (DNMTs) use CpG-rich regions as recognition cues to methylate cytosines (m5C), which suppresses transcription. Proteins with histone-binding domains that can “read,” “write,” or “erase” histone marks may detect the tails that protrude from histone octamers (Fanucchi et al., 2021). In addition to methylation, acetylation, phosphorylation, and ubiquitination, these enzymes can catalyze the addition or removal of a broad and diverse range of other histone modifications. Different DNA methylation patterns discriminate between “responders” (those who can experience taught immunity) and “non-responders” to stimuli, such as BCG, that produce trained immunity (Verma et al., 2017). Forty-three genes had distinct methylation patterns in BCG-naive responders as opposed to non-responders in a follow-up investigation, which may be utilized to predict sensitivity to triggers of trained immunity (Das et al., 2019). Numerous studies have shown that various DNA and histone modification combinations affect whether DNA is kept in an accessible or “open” state or an inaccessible or “closed” one (Fanucchi et al., 2021). To enable quick and effective transcriptional activation, highly accessible DNA is quickly bound by the transcriptional machinery and transcription factors. This establishes a clear connection between the transcriptional state of protein-coding genes and the “openness” of DNA.

#### 2.2.3 lncRNAs

During trained immunity, lncRNA-dependent regulation has a significant impact on the epigenetic reprogramming of immune genes (Fanucchi et al., 2021). Several lncRNAs known as immune-gene priming lncRNAs (IPLs) were found by the application of a bioinformatic pipeline that comprised 3D nuclear architecture, lncRNA and enhancer expression data, and the epigenetic status of immune genes at the genome scale (Fanucchi et al., 2019). The WD repeat-containing protein 5 (WDR5)-mixed lineage leukemia protein 1 complex is directed across the chemokine promoters by UMLILO (upstream master lncRNA of the inflammatory chemokine locus), allowing H3K4me3 epigenetic priming, according to careful analysis of a prototypical IPL known as UMLILO (Fanucchi et al., 2019). Several trained immune genes share this mechanism. Training mediated by β-glucan upregulates IPLs in a way that depends on the nuclear factor of activated T cells, which epigenetically reprograms immune genes. The Cxcl genes are not trained, and the murine chemokine topologically associating domain is devoid of an IPL. Cxcl genes are trained as a result of the insertion of UMLILO into the chemokine topologically associating domain in murine macrophages (Fanucchi et al., 2019). This offers compelling evidence that the development of trained immunity depends on lncRNA-mediated control. Further research is required to examine these pathways in various experimental contexts, as recent studies have only examined the function of IPLs in β-glucan-induced trained immune characteristics.

### 2.3 Modulation of myelopoiesis progenitors

The observation of trained circulating monocytes months after BCG vaccination suggests that adaptive processes induced by trained immunity involve alterations in hematopoietic progenitors at the bone marrow level (Kleinnijenhuis et al., 2012). Evidence shows that trained immunity plays a role at the bone marrow level in the context of AS. In mice, the administration of β-glucan leads to long-term transcriptional and metabolic alterations in hematopoietic stem and progenitor cells, resulting in their expansion and bias toward myelopoiesis. This enhances their ability to respond to secondary LPS stimulation and protects them from chemotherapy-induced myelosuppression (Mitroulis et al., 2018). The shared β-subunit of the IL-3/GM-CSF receptor, CD131, is linked with enhanced surface expression in this long-term reprogramming. In a mouse model of predisposition to AS, a similar process occurs when hypercholesterolemia induces enhanced myeloid proliferation and inflammation, suggesting a possible role for trained immunity in the context of traditional cardiovascular risk factors (Wang et al., 2014). Existing evidence also supports a link between enhanced glycolysis in myeloid cells and AS. In hypercholesterolemic ApoE^−/−^ mice, leucocytes and HSPCs show enhanced GLUT1-dependent glucose absorption, which is linked to an elevated mitochondrial potential, providing evidence for a role for myeloid cell glycolysis in myelopoiesis and atherogenesis. This suggests that the mitochondria in these cells are fed by the inflow of glycolytic metabolites for OXPHOS and ATP synthesis (Sarrazy et al., 2016).

## 3 Endogenous triggers of trained immunity

In addition to microbial sources, endogenous molecules, such as cellular metabolites oxLDL, lipoprotein(a), and hyperglycemia, can induce trained immunity (Bekkering et al., 2014; van der Valk et al., 2016; Braza et al., 2018; Edgar et al., 2021). These endogenous triggers play a role in the development of ASCVD (Flores-Gomez et al., 2021). We will discuss the link between these endogenous triggers of trained immunity and atherosclerotic plaque formation in activated monocyte–macrophages (Table 1).

**TABLE 1:** Endogenous triggers of trained immunity.

Ligand	Model	Receptor	Trained immunity signaling	Metabolic reprogramming	Epigenetic reprogramming	Atherogenic factor	Reference
oxLDL	Monocytes/macrophages	TLR	mTOR/HIF1-α	Glycolysis	H3K4me3	IL-6, TNF-α, SR-A, CD36, and MCP1	Bekkering et al. (2014); Sohrabi et al. (2018); Keating et al. (2020)
		IL-1		Mevalonate synthesis			
Lipoprotein(a)	Monocytes/macrophages	Oxidized phospholipids	—	—	—	IL-6 and TNF-α	van der Valk et al. (2016); Stiekema et al. (2020)
Hyperglycemia	BMHSCs	Runx1	IFN-γ	Glycolysis	H3K4me3 and H3K27ac	IL-6 and IL-1β	Edgar et al. (2021)

Catecholamines	BMHSCs	—	β-Adrenergic receptor 1 and 2 cAMP-protein kinase A	Glycolysis and oxidative phosphorylation	H3K4me3	IL-6, IL-8, and TNF-α	van der Heijden et al. (2020a)

Aldosterone	Monocytes/macrophages	Mineralocorticoid	Fatty acid synthesis pathway	Fatty acid synthesis	H3K4me3	Arterial wall inflammation	van der Heijden et al. (2020b); van der Heijden et al. (2020c)
Hyperlipidemia	BMHSCs	—	NLRP3	Cholesterol biosynthesis pathway	Chromatin landscape	—	Christ et al. (2018)
			IL-1				

Abbreviation: BMHSCs, bone marrow hematopoietic stem cells; Runx1, Runt-related transcription factor 1; HIF1α, hypoxia-inducible factor 1-alpha; oxLDL, oxidized low-density lipoprotein; IFN-γ, interferon-gamma; TLR, Toll-like receptor; mTOR, mammalian target of rapamycin; NLRP3, NLR family pyrin domain-containing 3; H3K4m3, histone 3 lysine 4 tri-methylation; H3K27ac, histone 3 lysine 27 acetylation; SR-A, type A scavenger receptor; CD36, cluster of differentiation 36; MCP1, monocyte chemoattractant protein 1; TNF-α, tumor necrosis factor-α; IL, interleukin.

### 3.1 oxLDL

oxLDL is a modified lipoprotein and is one of the key atherogenic molecules within plaques that activates immune cells (Moore and Tabas, 2011). oxLDL-trained macrophages exhibit significant metabolic and epigenetic rewiring, similar to BCG and β-glucan. The mammalian target of the rapamycin (mTOR)/HIF1-α signaling pathway is necessary for the upregulation of glycolysis and OXPHOS in oxLDL-induced cells (Keating et al., 2020). The increase in glycolysis and the proinflammatory phenotype in macrophages were avoided by pharmacological suppression of the mTOR pathway and the signaling molecules involved and by inhibiting glycolysis with 2-deoxyglucose (Sohrabi et al., 2018). Epigenetic reprogramming is another characteristic of oxLDL-trained macrophages. OxLDL interacts with the myeloid cell surface receptor cluster of differentiation 36 (CD36) as a damage-associated molecular pattern (DAMP) (Moore et al., 2013). The internalization and release of oxLDL into the cytoplasm may create cholesterol crystals, which activates the NOD-, LRR-, and pyrin domain-containing protein 3 (NLRP3) inflammasome and releases IL-1β and other proinflammatory cytokines, as well as a protracted inflammatory response (Sheedy et al., 2013). Promoters of genes encoding proinflammatory and proatherogenic cytokines and chemokines, such as IL-6, TNF-α, type A scavenger receptor (SR-A), and CD36, are more likely to have the activating histone modification H3K4me3 ^20^. OxLDL training was fully blocked by pharmacologically inhibiting histone methyltransferases, demonstrating that epigenetic alterations are what actually trained immunity by oxLDL (Bekkering et al., 2014).

### 3.2 Lipoprotein(a)

Lipoprotein(a) is the main circulating carrier of oxidized phospholipids, which plays an important role in atherogenesis (Boffa and Koschinsky, 2019). Monocytes from healthy donors exposed for 24 h to high lipoprotein(a) extracted from hyperlipidemic patients produced more proinflammatory cytokines during the subsequent 6 days compared to controls. Anti-oxidized phospholipid antibodies reduced the training of monocyte-derived macrophages, demonstrating that oxidized phospholipids are the mediating factor in this process (van der Valk et al., 2016). After Pam3Cys and LPS *ex vivo* stimulation, monocytes showed an increased ability to generate proinflammatory cytokines, including IL-6 and TNF-α (van der Valk et al., 2016). A recent study has shown that individuals with cardiovascular diseases (CVD) may have their proinflammatory monocyte activation reversed by significantly reducing their lipoprotein(a) levels, demonstrating that at least some of this proinflammatory impact is reversible (Stiekema et al., 2020).

### 3.3 Hyperglycemia

Hyperglycemia, a cardinal feature of diabetes, exacerbates AS progression, delays plaque regression (Parathath et al., 2011), and increases proinflammatory gene expression and resistance to induction of M2-related gene expression (American Diabetes Association, 2015). Evidence suggests that hyperglycemia induces trained immunity in HSCs and macrophages, significantly exacerbating AS (Edgar et al., 2021). High extracellular glucose stimulated the production of proinflammatory genes and the functional properties that are proatherogenic in macrophages through pathways that depend on glycolysis. These traits were sustained by diabetic mouse bone marrow-derived macrophages even when they were cultivated in physiological glucose, showing hyperglycemia-induced trained immunity. A disease-relevant and enduring kind of trained innate immunity was demonstrated by an increase in aortic root AS following bone marrow transplantation from diabetic mice into (normoglycemic) Ldlr^−/−^ mice. HSCs and macrophages generated from the bone marrow showed a proinflammatory priming effect in diabetes, according to integrated tests for transposase-accessible chromatin, chromatin immunoprecipitation, and RNA sequencing analysis (Edgar et al., 2021). Transcription factors, notably runt-related transcription factor 1 (Runx1), are implicated as mediators of trained immunity (Himes et al., 2005). These *in vitro* signs of trained immunity brought on by hyperglycemia were eliminated by pharmacological suppression of Runx1.

### 3.4 Catecholamines

Increased sympathetic nervous system activity leads to proinflammatory leukocytosis in models of chronic psychological stress, stroke, and myocardial infarction (Dutta et al., 2012; Heidt et al., 2014; Courties et al., 2015). The pathways causing inflammatory alterations in disorders with high catecholamine levels can be explained by the fact that catecholamines cause long-lasting proinflammatory modifications in monocytes *in vitro* and *in vivo*, indicating well-trained immunity (van der Heijden et al., 2020a). After being restimulated with LPS 6 days later, monocyte-derived macrophages exposed to a relevant quantity of epinephrine/norepinephrine had higher levels of TNF-α and IL-6. Similar to oxLDL, this trained immune phenotype is connected to a higher glycolytic capability and OXPHOS. Studies using pharmacological inhibition demonstrated that the cAMP-protein kinase A pathway and the β-adrenergic receptors 1 and 2 are crucial for catecholamine-induced training (van der Heijden et al., 2020a). Patients who have pheochromocytoma and are regularly exposed to brief bursts of catecholamine production have this proinflammatory monocyte characteristic (Neumann and Young, 2019). Systemic inflammatory symptoms and an increased *ex vivo* cytokine response in activated monocytes were present in these individuals (Neumann and Young, 2019).

### 3.5 Aldosterone

Human macrophages deriving from monocytes have a long-lasting proinflammatory phenotype *in vitro* in response to transiently elevated aldosterone concentrations, which may be a factor in the atherosclerotic condition AS chronic inflammation of the artery wall (van der Heijden et al., 2020d). Aldosterone affects intracellular metabolism by increasing fatty acid synthesis, but it does not influence glycolysis and OXPHOS, as found in oxLDL training (van der Heijden et al., 2020c). Additionally, training by aldosterone is linked to the enrichment of H3K4me3 at the promoters of proinflammatory cytokines, including TNF-α and IL-6, demonstrating that aldosterone trains monocyte-derived macrophages *in vitro*. However, circulating monocytes are not more capable of producing cytokines in individuals with primary hyperaldosteronism. The macrophages of individuals with primary hyperaldosteronism only express more TNF-α following *ex vivo* differentiation into macrophages in autologous serum (van der Heijden et al., 2020b). These findings imply that aldosterone differs from the trained immune systems that have been well-established and elaborated by other stimuli.

### 3.6 Hyperlipidemia

Recent research examined the possibility that a WTD, which is high in fats, sweets, and salt and lacks fiber, might lead to trained immunity (Christ et al., 2018). Circulating monocytes and their myeloid progenitors in AS-prone Ldlr^−/−^ mice were significantly affected by proinflammatory transcriptional and epigenetic reprogramming from this WTD over 4 weeks. Increased inflammatory responses to subsequent innate immunological stimulation were brought on by the food intervention. Even when the mice were shifted to a typical chow diet for an additional 4 weeks, this trained immune phenotype was maintained despite circulating cholesterol levels and systemic inflammatory indicators reverting to normal (Christ et al., 2018).

## 4 Inhibitors of targeting trained immunity

Pharmacological inhibitors of histone methyltransferases and inhibitors of glycolysis, glutaminolysis, and the mevalonate pathway could restrain trained immunity. Following intraperitoneal injection of β-glucan, the effects of pharmacologically suppressing glutaminolysis and the pathway that produces mevalonate on the formation of trained immunity have been established in mouse models *in vivo* (Arts et al., 2016a). As a result, it would allow the development of innovative pharmaceutical methods to lower the risk of ASCVD and maybe lessen its negative consequences. In this section, we systematically summarize all reported drugs that suppress trained immunity based on multiple publications.

### 4.1 Agents that modulate metabolic reprogramming

#### 4.1.1 Wortmannin

The fungus metabolite wortmannin was demonstrated to function as a selective inhibitor of AKT/phosphoinositide 3-kinase (PI3K) (Ui et al., 1995). The intermediate stimulation of the Akt/PI3K pathway is what causes mTOR to become active (Kelley et al., 1999). As stimulation with β-glucan caused a high phosphorylation of Akt, β-glucan was responsible for inducing this signal pathway in monocytes. Additionally, mTOR activation was inhibited as a result of Akt phosphorylation inhibition. Monocyte-trained immunity by β-glucan was suppressed by the Akt inhibitor wortmannin in a dose-dependent manner (Cheng et al., 2014).

#### 4.1.2 Rapamycin

Accumulated mevalonate enhances the AKT-mTOR pathway during the establishment of trained immunity, which then triggers HIF1-α activation and a switch from OXPHOS to glycolysis. This response results in circulating monocytes with a trained immunity phenotype (Bekkering et al., 2018). The inhibition of mTOR with rapamycin prevents mevalonate-induced trained immunity. Additionally, BCG-induced trained immunity and β-glucan-induced trained immunity depend on the development of the histone marks H3K4me3 and H3K9me3, which are inhibited by the pharmacological regulation of rate-limiting glycolysis enzymes with rapamycin ^30 88^. Although rapamycins potently suppress trained immunity *in vitro* and T-cell proliferation *in vivo*, they exert little effect on innate immune cells (Braza et al., 2018).

#### 4.1.3 AICAr

One of the most widely utilized pharmacological AMP-activated protein kinase (AMPK) activity modulators is the nucleoside 5-aminoimidazole-4-carboxamide (AICAr). Early research on AMPK’s function in the physiological control of metabolism and the etiology of cancer was mostly centered on the use of AICAr as an AMPK activator (Višnjić et al., 2021). AICAr produces dose-dependent inhibition of β-glucan-induced trained immunity by indirectly inhibiting mTOR (Cheng et al., 2014).

#### 4.1.4 Metformin

Metformin is extensively used as a first-line therapy for type 2 diabetes and has a high safety profile (McCreight et al., 2016). Metformin acts through AMPK activation and subsequent mTOR inhibition. Metformin completely inhibits the protective effects of mice receiving metformin during and after primary infection with low-inoculum *C. albicans*, which increases survival during disseminated candidiasis brought on by a primary *C. albicans* injection. *In vitro*, metformin suppresses trained immunity induced by β-glucan (Cheng et al., 2014). Metformin also inhibits trained immunity by inhibiting the formation of histone marks, H3K4me3 and H3K9me3, by regulating the rate-limiting enzymes of glycolysis ^30 88^.

#### 4.1.5 Ascorbate

Ascorbate (vitamin C) is an essential micronutrient in primates and serves as an antioxidant and a cofactor for various enzymatic activities represented by prolyl hydroxylases (Fujii et al., 2022). Because the induction of glycolysis by mTOR is mediated by the activation of HIF1-α and stimulation of glycolytic enzymes and ascorbate inhibits HIF-1α expression, it inhibits training immune in a dose-dependent manner (Cheng et al., 2014).

#### 4.1.6 ZVAD-fmk

Western diet feeding of Ldlr^−/−^ mice induces systemic inflammation, which induces long-lasting trained immunity in myeloid cells (Christ et al., 2018). NLRP3 is a key pathway mediating Western diet-induced trained immunity, and the use of small-molecule inhibitors that block NLRP3 signaling can mitigate its potentially deleterious effects in inflammatory diseases (Christ et al., 2018). ZVAD-fmk (benzyloxycarbonyl-Val-Ala-Asp-fluoromethylketone), a pan-caspase inhibitor, inhibits NLRP3 inflammasome activation in atherosclerotic mice, reducing the accumulation of serum IL-1β and plaque cholesterol crystals (Sheedy et al., 2013).

#### 4.1.7 2-DG

A d-glucose mimic, 2-deoxy-d-glucose (2-DG), inhibits glycolysis by producing and accumulating intracellularly 2-deoxy-d-glucose-6-phosphate (2-DG6P), which then inhibits the activity of hexokinase and glucose-6-phosphate isomerase and results in cell death (Pajak et al., 2019). BCG immunization causes immunometabolic activation and epigenetic reprogramming, whereas 2-DG’s restriction of glycolysis during BCG-induced training cancels out the enhanced cytokine production (Arts et al., 2016b). In addition, the inhibitory effect of 2-DG on glycolysis also inhibits histone methylation and prevents mevalonate-induced trained immunity (Arts et al., 2016b).

#### 4.1.8 3PO

3-(3-Pyridinyl)-1-(4-pyridinyl)-2-propen-1-one (3PO), a small-molecule inhibitor of PFKFB3, inhibits glycolytic flow and is cytostatic to malignant cells (Clem et al., 2008). In cells trained with oxLDL, PFKFB3, a critical rate-limiting enzyme in glycolysis, is increased. The *in vitro* training protocol’s dose-dependent attenuation of the oxLDL-augmented production of TNF-α and IL-6 upon subsequent stimulation with LPS was achieved by co-incubating 3PO with oxLDL for the first 24 h (Clem et al., 2008).

#### 4.1.9 Fluvastatin

Fluvastatin, the first fully synthetic HMG-CoA reductase (HMGCR) inhibitor, is reported to prevent the growth and spread of certain malignancies (Cai and Zhao, 2021). Fluvastatin prevents trained immunity by downregulating H3K4me3 and blocking the production of proinflammatory cytokines (Arts et al., 2016a). Fluvastatin also prevents the enhanced foam cell production brought on by training with oxLDL and stops the epigenetic reprogramming of BCG, β-glucan, and oxLDL-induced trained immunity (Arts et al., 2016a). Additionally, following OxLDL-induced trained immunity, scavenger receptor CD36 and SR-A mRNA expression increase, whereas cholesterol efflux transporter ATP binding cassette transporter A1 (ABCA1) and ATP binding cassette transporter G1 (ABCG1) decrease. These effects may be reversed by adding fluvastatin (Bekkering et al., 2018).

#### 4.1.10 Cerulenin

Cerulenin is a potent and specific inhibitor of type II FAS found in various bacteria and mammalian tissues (Tomoda et al., 1984). It is an antifungal antibiotic discovered in a culture filtrate of *Cephalosporium caerulens* (Porrini et al., 2014). Aldosterone levels above normal are linked to a higher risk of CVD in people and the induction of trained immunity in primary human monocytes (van der Heijden et al., 2019). Aldosterone’s trained immunity was reduced when cells were pre-incubated with the fatty acid synthesis inhibitor cerulenin for 1 h before re-stimulating with P3C (van der Heijden et al., 2019).

### 4.2 Agents that modulate epigenetic reprogramming

#### 4.2.1 Ro5-3335

Extracellular glucose promotes macrophage-trained immunity and induces a pro-atherogenic phenotype through a glycolysis-dependent pathway. Runx1, which mediates trained immunity produced by hyperglycemia, is implicated by the pattern of open chromatin (Edgar et al., 2021). A benzodiazepine identified from the screen, Ro5-3335, has a direct interaction with Runx1 (Cunningham et al., 2012). *In vitro* hyperglycemia-induced trained immunity was reversed by pharmacological suppression of Runx1 with Ro5-3335 ^4^.

#### 4.2.2 MTA

The histone methyltransferase inhibitor 5′-deoxy-5′-methylthioadenosine (MTA) is a non-selective methyltransferase inhibitor. OxLDL causes monocytes to develop a proinflammatory phenotype that persists over time and speeds up AS. MTA completely reverses the methylation of histones, which is required for the change in chromatin architecture that results in increased gene transcription, and thus completely reverses the trained immunity phenotype induced by oxLDL (Bekkering et al., 2014).

#### 4.2.3 Resveratrol

Sirtuin 1 is a nicotinamide adenine dinucleotide (NAD^+^)-dependent protein deacetylase and master metabolic regulator (Deng et al., 2019). Phytochemical resveratrol, which is abundant in the skin of red grapes and wine, has been studied extensively for its ability to stimulate Sirtuin 1 activity (Lee et al., 2019). Given that histone acetylation is necessary for β-glucan-induced trained immunity, trained immunity in the presence of the histone deacetylase activator resveratrol prevented trained SHIP-1-deficient macrophages from producing more TNF-α (Saz-Leal et al., 2018).

#### 4.2.4 EGCG

The compound epigallocatechin-3-gallate (EGCG) has been discovered to be a new histone acetyltransferase inhibitor (HATi) with broad specificity for the majority of HAT enzymes (Choi et al., 2009). EGCG can also inhibit trained immunity that relies on β-glucan-induced epigenetic reprogramming (Ifrim et al., 2014).

## 5 Antiatherosclerotic herbal medicine potentially targeting trained immunity

The notion that trained monocytes/macrophages exhibit a broad range of pro-atherogenic phenotypes, including increased production of cytokines/chemokines and foam cells, has recently been extensively supported experimentally (Leentjens et al., 2018). Trained immunity occurs not only in circulating monocytes but also in myeloid progenitors, ensuring a long-term state of hyperactivation of innate immune cells. Trained immunity is mediated by metabolic and epigenetic reprogramming at the level of histone methylation. Theoretically, these processes are amenable to pharmacological intervention. In the past few decades, more studies have shown that various naturally occurring anti-atherogenic natural products, such as flavonoids, phenols, terpenoids, carotenoids, phenylpropanoids, and alkaloids, may be involved in the regulation of pharmacological targets of trained immunity (Supplementary Table S2). We systematically summarize all relevant literature to investigate all potential natural products against trained immunity in ASCVD.

### 5.1 Flavonoids

Flavonoids are a group of secondary plant metabolites often employed by vegetables for growth and microbial defense (Izzo et al., 2020). Flavonoids can be further classified as flavones, flavonols, flavanones, isoflavonoids, anthocyanins, flavanols, or catechins based on structural distinctions (Kumar and Pandey, 2013). Due to their antioxidant, anti-inflammatory, anti-mutagenic, anti-aging, cardioprotective, antiviral/bacterial, and anticarcinogenic qualities and their ability to influence enzyme performance, they are linked to a variety of positive health impacts. Flavonoids are thought to mediate epigenetic changes, including DNA methylation, histone modifications, and non-coding RNAs (Fatima et al., 2021). We examine some significant natural products that may target monocyte/macrophage and trained immunity in AS in this section.

#### 5.1.1 Alpinetin

Alpinetin (7-hydroxy-5-methoxyflavanone), a flavonoid, is the main active component of *Alpinia katsumadai* Hayata, a traditional medicinal plant. It engages in various biological processes that affect the NF-κB, MAPK, and PI3K signaling pathways, such as antibacterial, anti-ROS, anticancer, and anti-inflammatory actions (Huo et al., 2012; Wu et al., 2020a; Zhang et al., 2020). The inhibition of the NLRP3 inflammasome may be one way of suppressing trained immunity (Christ et al., 2018). Mechanistically, alpinetin inhibits NLRP3-mediated anti-inflammatory activity and reduces mitochondrial ROS production and HIF-1α transcription, thereby inhibiting HIF-1α signaling (Zhang et al., 2020; Zhu et al., 2021). The expression of the toll-like receptor 4 (TLR4) stimulated by LPS may be dramatically downregulated by alpinetin; alpinetin was reported to have had an anti-inflammatory impact by preventing the production of TNF-α, IL-6, and IL-1β in LPS-stimulated human macrophages (Hu et al., 2013).

#### 5.1.2 Anthocyanins

Anthocyanins are water-soluble glycosides of polyhydroxyl and polymethoxyl derivatives of 2-phenylbenzopyrylium or flavylium salts and are partially responsible for the pigmentation of berries (Azzini et al., 2017). The major anthocyanins in plant foods are glycoside forms of anthocyanidins, including pelargonidin, cyanidin, delphinidin, peonidin, petunidin, and malvidin (Khoo et al., 2017). The bioavailability of anthocyanins is higher than previously thought because the parent compounds are immediately absorbed and converted to bioactive metabolites that remain in circulation (Scalbert et al., 2005; Czank et al., 2013). Anthocyanins increase total antioxidant capacity, antioxidant defense enzymes, and high-density lipoprotein (HDL) antioxidant properties in preclinical and clinical populations through multiple measures, thereby reducing CVD risk factors and mortality in patients with coronary heart disease (Garcia and Blesso, 2021). An essential mediator of trained immunity, the NLRP3-caspase-1 inflammasome, is directly activated upstream by ROS, which is an important mediator of trained immunity (Sun et al., 2020). Preclinical research suggests that anthocyanidins regulate cellular cholesterol efflux from macrophages, hepatic paraoxonase 1 expression, and activity to affect reverse cholesterol transport (RCT) and HDL function beyond simple HDL cholesterol content (Millar et al., 2017). In human populations (such as those who are hyperlipidemic, hypertensive, or diabetic), dietary anthocyanin intake is linked to positive changes in serum biomarkers related to HDL function. These changes include an increase in HDL cholesterol concentration and HDL antioxidant and cholesterol efflux capacities (Millar et al., 2017).

The powdered wild blueberry (*Vaccinium angustifolium*) component high in anthocyanins also reduced lipid buildup in macrophages generated from THP-1 (Del Bo et al., 2016). In accordance with additional studies, the black rice anthocyanin-rich extract blocked the generation of oxLDL and decreased total cholesterol (TC) and LDL-cholesterol (LDL-C) while boosting the amount of HDL-cholesterol (HDL-C) in serum from rats and ApoE^−/−^ mice. In order to lower the risk of an embolism, it also decreased the area of atherosclerotic plaque and improved the stability of the plaque (Xia et al., 2006). In hypercholesterolemic rabbits, fatty streak development and lipid metabolism were slightly influenced by pomegranate peel extract containing anthocyanins (Sharifiyan et al., 2016). This evidence suggests that the potential of anthocyanins to regulate inflammation, lipid buildup, and macrophage may play a role in how an anthocyanin-rich diet lowers the risk of developing CVD.

##### 5.1.2.1 Cyanidin-3-O-β-glucoside

Cyanidin-3-O-glucoside (C3G) is the anthocyanin with the greatest abundance. C3G is abundantly found in fresh fruits, including grapes, berries, blood oranges, peaches, and apples, and in beverages and colored cereals, such as purple rice and maize (Fang, 2015). One investigation found that methylated proteins, particularly H3K4, lose mono- or dimethyl groups when exposed to C3G or its metabolites, which block the enzyme lysine-specific demethylase 1, which controls histone methylation (Abdulla et al., 2013), thereby directly affecting histone-modifying enzymes (Persico et al., 2021). The results reported here show how dietary C3G intake may effectively control H3K4me3 in the mouse liver, especially in promoter areas (Persico et al., 2021). Recent research in a rat model of high-fat diet (HFD)-induced AS examined the antiatherosclerotic potential of C3G. The findings demonstrated that adding 150 mg/kg of C3G to the diet significantly reduced body weight, visceral adiposity, TG, TC, free fatty acids, and AS index (Um et al., 2013). C3G protected ApoE^−/−^ mice against endothelial dysfunction and AS brought on by hypercholesterolemia by preventing the buildup of cholesterol and 7-oxysterol in the aorta (Wang et al., 2012).

#### 5.1.3 Baicalin

Baicalin is a flavonoid active ingredient extracted from the roots of *Scutellaria baicalensis* Georgi, a plant used for many years in Chinese traditional medicine to treat various inflammatory illnesses (Liu and Liu, 2017; Riham et al., 2019). One study showed that baicalin could control metabolic diseases *in vivo*. The therapy with baicalin in HFD rats markedly improved fasting blood glucose levels (Guo et al., 2009). Furthermore, baicalin is reported to inactivate succinate dehydrogenase (SDH) to inhibit ROS production and protect glutamine synthetase (GS) protein stability from oxidative stress to improve glutamate handling and reduce excitotoxicity (Song et al., 2020a).

#### 5.1.4 Chrysin

Chrysin (5,7-dihydroxyflavone) is a flavonoid that naturally occurs in food and is frequently found in honey and propolis, among other plant extracts (Song et al., 2020b). Chrysin possesses various biological qualities, including anti-inflammatory, anti-bacterial, antidiabetic, anticancer, antioxidant, and anti-allergenic actions (Kasala et al., 2015; Mani and Natesan, 2018). One study showed that chrysin has a good expansion effect on human HSCs due to its antioxidant properties by delaying HSC differentiation, inhibiting ROS-activated apoptosis, and regulating cyclin-dependent kinase inhibitors, which can maintain the self-renewal and multilineage differentiation potential of human HSCs (Litviňuková et al., 2020). Chrysin is an emerging histone deacetylase inhibitor for epigenetic regulation in cancer studies (Ganai et al., 2021).

Chrysin may reduce inflammation by modulating M1/M2 status. It promotes the anti-inflammatory M2 phenotype and suppresses the M1 phenotype in peritoneal and cultured macrophages *in vitro* by activating PPAR-γ (Feng et al., 2014). One study showed that chrysin inhibited NLRP3 inflammasome activation and increased IL-1β levels to reduce synovitis (Liao et al., 2020). Another study showed that chrysin inhibits ROS-mediated Akt/mTOR signaling in cells and induces autophagy (He et al., 2021). The study showed that the overexpression of PPARγ, liver X receptor (LXR)α, ABCA1, and ABCG1 expression led to a considerable increase in HDL-mediated RAW264.7 macrophage cholesterol efflux following chrysin treatment (Lin et al., 2015b).

#### 5.1.5 Daidzein

Daidzein, a substance mostly present in soy foods and plants such as red clover, is one of the most studied and potent phytoestrogens (Yang et al., 2012). Studies in non-human primates have shown that dietary intake of soy protein can interfere with related epigenetic changes that may influence the etiology of complex diseases (Howard et al., 2011). By stimulating the PPARγ-LXRα-ABCA1 pathway, daidzein protected low-density lipoprotein (LDL) from oxidation and increased paraoxonase-1 (PON-1) activity in Huh7 cells, which may control cholesterol efflux (Schrader et al., 2012; Ikhlef et al., 2016). Furthermore, daidzein therapy decreased blood cholesterol and increased TG levels in middle-aged male rats given HFD designed to induce AS (Sosić-Jurjević et al., 2007). These suggest that daidzein has antiatherosclerotic potential.

#### 5.1.6 Ellagic acid

Ellagic acid (EA) is a dilactone of hexahydroxydiphenic acid that may be found in various nuts, fruits, and vegetables, such as pomegranates, walnuts, black raspberries, raspberries, almonds, and strawberries (Galano et al., 2014). According to *in vitro*, *in vivo*, and clinical investigations, it has a wide range of physiological actions, including anti-inflammatory, antioxidant, antibacterial, anticarcinogenic, antiplasmodial, antiviral, hepatoprotective, antifibrotic, immunomodulatory, and neuroprotective activities (Gupta et al., 2021). A study showed that EA promotes hematopoietic progenitor cell proliferation and megakaryocyte differentiation (Gao et al., 2014). Other studies have shown that EA interrupts the sequential histone remodeling steps of adipocyte differentiation by reducing the coactivator-associated arginine methyltransferase 1 (CARM1) activity, including histone acetylation and dissociation of HDAC9 from chromatin (Kang et al., 2014). Pomegranate peel polyphenols, in particular pomegranate ellagic acid (PEA), also boosted ApoA1-mediated macrophage cholesterol efflux by upregulating ABCA1 and LXRα and inhibited macrophage lipid buildup by lowering the expression of CD36 (Zhao et al., 2016).

#### 5.1.7 EGCG

EGCG, a typical polyphenol flavonoid molecule with eight free hydroxyl groups, is the most common (Chakrawarti et al., 2016). Research has revealed that EGCG has antibacterial, antiviral, antioxidant, anti-arteriosclerosis, anti-thrombosis, anti-vascular proliferation, anti-inflammatory, and anti-tumor activities (Liu and Yan, 2019). As a histone acetyltransferase inhibitor, EGCG can significantly inhibit the training of monocytes (Ifrim et al., 2014). In different research, EGCG-loaded nanoparticles targeted macrophages *via* their CD36 receptor, reduced the release of inflammatory factors by mouse peritoneal macrophages, and reduced the lesion surface area of arterial plaques in LDLr^−/−^ mice (Zhang et al., 2019). EGCG also inhibited the oxLDL-induced overexpression of SR-A in the same cell line, reducing oxLDL absorption and the formation of foam cells (Chen et al., 2017). EGCG regulated macrophage polarization toward the M2 state. EGCG decreased the expression of proinflammatory M1 mediators, iNOS, TNF-α, IL-1β, and IL-6, in the LPS-administered lung microenvironment and increased the expression of KLF4, Arg1, and ym1, which enhanced the M2 phenotype of macrophages (Almatroodi et al., 2020).

#### 5.1.8 Hesperidin

Hesperidin (3′,5,7,-trihydroxy-4′-methoxyflavanone), a flavanone family of flavonoids, is a derivative of hesperetin, which is present in citrus fruits, such as oranges and grapefruit (Muhammad et al., 2019). Hesperidin has several pharmacological effects, with the main ones being the stimulation of antioxidation, the inhibition of the generation of proinflammatory cytokines, and the inhibition of the proliferation of cancer cells (Li and Schluesener, 2017). A study using metabolic tracing studies showed that TLR signaling in mouse and human macrophages redirects metabolic flux to increase acetyl-CoA for glucose production, thereby enhancing histone acetylation (Christ and Latz, 2019). According to a preclinical study, hesperetin provided neuroprotection by controlling the TLR4/NF-κB signaling pathway in response to the harmful effects of LPS (Muhammad et al., 2019). Hesperetin decreased the generation of foam cells produced from THP-1 by promoting ABCA1 expression by boosting the activities of the ABCA1 promoter and LXR enhancer, upregulating the ApoA1-mediated cholesterol efflux (Iio et al., 2012).

#### 5.1.9 Icariin

Icariin, one of the primary ingredients in epimedium, is an 8-isopentane flavonoside that has several pharmacological benefits, including enhancement of cardiovascular function, promotion of hematological function, prevention of neuronal damage, and anti-osteoporosis properties (El-Shitany and Eid, 2019). Icariin decreased RAW264.7 macrophage infiltration at atherosclerotic lesions by reducing the CX3CR1–CX3CL1 interaction, which is directly related to monocyte adhesion and migration (Wang et al., 2016a).

#### 5.1.10 Pratensein

Pratensein is a compound extracted from Radix Polygala roots. It has anti-inflammatory, anti-apoptotic, and antioxidant effects (Liu et al., 2016b). Pratensein increases the expression of the ABCA1 protein and HDL levels in HepG2 cells (Gao et al., 2008). AS is brought on by passive LDL transport across damaged endothelial cells. Recent research has revealed a novel therapeutic target in the fight against AS: scavenger receptor class B type I (SR-BI)-mediated endothelial LDL transcytosis. This process increases LDL entry into the arterial wall and the development of AS (Huang et al., 2019). Further investigation found that pratensein increased the expression of CLA-1, a human homolog of SR-BI, indicating that it may have some bearing on the *in vitro* process of cholesterol efflux (Yang et al., 2009).

#### 5.1.11 Puerarin

Puerarin, an isoflavone component extracted from the herb Radix Puerariae, is often employed in China to treat inflammatory and immunological disorders (Yang et al., 2021). Its powerful pharmacological effects are a result of the compounds’ many bioactivities. Puerarin’s anti-inflammatory processes, which include the control of important signals, including TLR, Nrf2, HDAC, and PPARα, and the enhancement of organelle function (Ni et al., 2020; Niu et al., 2020; Chen et al., 2021), have been thoroughly investigated in recent years (Chang et al., 2021). An earlier study investigated the epigenetic mechanism through which puerarin suppresses MCP-1 production using high-glucose circumstances. It was shown that puerarin dramatically reduced high glucose’s ability to upregulate H3K4 di- and tri-methylation (H3K4me2/3) on the MCP-1 gene promoter, suggesting that it may be useful in treating diabetes-related vascular damage (Han et al., 2015). Puerarin promoted ABCA1-mediated cholesterol efflux *via* pathways involving miRNA-7, serine/threonine kinase 11 (STK11), and the AMPK-PPARγ-LXRα-ABCA1 cascade, therefore reducing cellular lipid buildup in THP-1 macrophages (Li et al., 2017a).

#### 5.1.12 Quercetin

Quercetin (3,3′,4′,5,7-pentahydroxyflavone) is one of the most prevalent plant flavonoids and a key dietary antioxidant in the human diet (Boots et al., 2008). It can be found in various traditional Chinese herbal medicines, tea, fruit, and other vegetables and has also been proven effective in clinical studies (Ferry et al., 1996). The antioxidant, anti-inflammatory, antiviral, anticancer, and antifibrotic effects of quercetin should be preserved (Russo et al., 2012). Moreover, quercetin stimulates autophagy in the hematopoietic stem/progenitor cell compartment of myelodysplastic bone marrow (Daw and Law, 2021). Qu also promotes apoptosis through DNA demethylation activity, HDAC inhibition, and enrichment of H3ac and H4ac in the promoter regions of genes that enhance apoptotic pathways (Alvarez et al., 2018).

*In vitro* studies have shown that quercetin can inhibit two stages of macrophage differentiation and polarization: macrophage infiltration (from monocytes to macrophages) and macrophage subtype conversion (from M2 to M1 subtypes). Quercetin downregulated the expression of M1 macrophage markers and proinflammatory cytokines and upregulated the expression of M2 macrophage markers and anti-inflammatory cytokines in BMDM under both basal and LPS-stimulated conditions (Dong et al., 2014b). Jia et al. (2019) demonstrated that in apoE^−/−^ mice fed with HFD, quercetin protects against AS by regulating the expression of proprotein convertase subtilisin/kexin type 9 (PCSK9), CD36, PPARγ, LXRα, and ABCA1. In THP-1-derived foam cells, quercetin increased ApoA1-mediated cholesterol efflux and promoted ABCA1 and PPARγ expression by activating PPARγ signaling (Sun et al., 2015). Quercetin has also been linked to reduced AS in ApoE^−/−^ mice by enhancing RCT, which depends on ABCA1 and ABCG1 (Cui et al., 2017).

#### 5.1.13 Silymarin

Silymarin is extracted from the seeds of *Silybum marianum* L. Gaertn. (also known as milk thistle). Silymarin is a blend of flavonoids, primarily silybin, silydianin, silychristin, and other active components (Rašković et al., 2011). In addition to protecting the liver and lowering enzymes and lipids, this combination has antioxidant, anti-inflammatory, and anticancer properties (Zhao et al., 2021). Studies have shown that silibinin may interfere with epigenetic cellular mechanisms, including increasing the total DNMT activity, while reducing histone deacetylase (HDAC) expression levels (Anestopoulos et al., 2016). The silymarin compounds, isosilybin A, isosilybin B, silychristin, and isosilychristin, increased ABCA1 protein expression in THP-1 cells. Due to its PPARγ activating qualities, isosilybin A, in particular, enhanced cholesterol efflux from THP-1 macrophages (Wang et al., 2015b).

### 5.2 Phenols

Phenolic chemicals are found all over the plant world and have more than 8,000 distinct known structures. Phenols can be classified as monophenols, binary phenols, or polyphenols, depending on the number of phenolic hydroxyl groups in the chemical structure (Xiao et al., 2012). Through various mechanisms, phenolic compounds have various pharmacological and biological actions. These activities include the control of various cell signaling pathways, gene expression, and antioxidation.

#### 5.2.1 Curcumin

Curcumin ((1E, 6E)-1,7-bis(4-hydroxy-3-methoxyphenyl)-1,6-heptadiene-3,5-dione) is a polyphenolic derivative produced from turmeric (*Curcuma longa*) (Wu et al., 2020b). Curcumin can control inflammation in *in vitro* and *in vivo* studies. This property makes curcumin an effective treatment for various inflammatory disorders, including obesity, diabetes, CVD, bronchial asthma, and rheumatoid arthritis (Chen et al., 2019a). Studies have demonstrated a direct inhibitory effect of curcumin on NLRP3 inflammasome activation in macrophages, which can prevent HFD-induced insulin resistance and inhibit LPS-priming and NLRP3 inflammasome activation pathways in macrophages (Yin et al., 2018). Curcumin may also act as an epigenetic regulator, including the inhibition of DNMTs, regulation of histone modifications *via* the regulation of histone acetyltransferases (HATs) and HDACs, regulation of miRNA, action as a DNA-binding agent, and interaction with transcription factors (Hassan et al., 2019). Additionally, c-Jun N-terminal kinases (JNK), histone methyltransferase p300, and transcriptional factor activating protein-1 (AP-1) were all inhibited by curcumin (Huwait et al., 2011).

In terms of regulating M1/M2 macrophages, curcumin can enhance the secretion of M2 macrophage markers, such as macrophage mannose receptor (MMR), Arg-1, PPAR-γ, IL-4, and/or IL-13. These effects have been observed in experimental autoimmune myocarditis (EAM) models and hyaline membrane disease, where curcumin polarizes M0 and M1 macrophages to an M2 phenotype (Saqib et al., 2018). A previous study indicated that without having a sizable impact on other cholesterol transporters, curcumin dramatically reduced the oxLDL-induced lipid buildup in J774.A1 macrophages by reducing the SR-A-dependent oxLDL uptake and enhancing the ABCA1-dependent cholesterol efflux (Zhao et al., 2012). The evidence suggests that curcumin inhibits the production of SR-A through a ubiquitin/proteasome mechanism. Additionally, through LXRα-dependent transcriptional regulation, curcumin increased ABCA1 expression (Zhao et al., 2012).

#### 5.2.2 Paeonol

Paeonol is an active component of the Chinese herbal remedy Moutan Cortex (Pae, 2-hydroxy-4-methoxyacetophenone), which is obtained from the root bark (Chen et al., 2019b). Pae has several physiologic benefits, including anti-inflammatory and antioxidant properties (Mei et al., 2019). LncRNAs are RNA molecules longer than 200 nucleotides interacting with target genes at the transcriptional level. They function as competing endogenous RNA (ceRNA) sponges to control mRNA expression and promote cisplatin-induced nephrotoxicity by regulating AKT/TSC/mTOR-mediated autophagy (Jing et al., 2021). Pae can inhibit the expression of lnc-MEG3 to alleviate renal injury in mice (Jing et al., 2021).

In ApoE^−/−^ mice, Pae therapy decreased the development of atherosclerotic lesions, slowed systemic inflammation, and enhanced ABCA1 expression (Zhao et al., 2013b).

#### 5.2.3 Polydatin

The main active ingredient of *Polygonum cuspidatum* Sieb. et Zucc. (Polygonaceae), a plant widely used in traditional medicine worldwide, particularly in China and Japan, is polydatin (Du et al., 2013). Polydatin clearly possesses hypoglycemic, antiatherosclerotic, hypolipidemic, hypouricemic, and anti-inflammatory effects, according to the findings of the literature review (Luo et al., 2022). In oxLDL-stimulated ApoE^−/−^ mouse macrophages, a 48 h polydatin therapy decreased TC, FC, and CE levels and TNF-α and IL-1β production. The mechanism generating these effects is linked to the stimulation of PPARγ-dependent ABCA1 overexpression and the decrease in CD36 expression (Wu et al., 2015a).

#### 5.2.4 Protocatechuic acid

In vegetables, fruits, and rice, protocatechuic acid (PCA, 3,4-dihydroxybenzoic acid) has been demonstrated to enhance vasodilation in apolipoprotein E-deficient rats with AS *via* the eNOS-mediated endothelium-dependent pathway (Bai et al., 2021). PCA may also control lipid metabolism by inhibiting the expression of HMG-CoA reductase (Liu et al., 2010). PCA also prevent ED intercellular adhesion molecule 1 (ICAM-1) and vascular cell adhesion molecule 1 (VCAM-1)-dependent monocyte adherence to activated HUVECs as well as CCL2-mediated monocyte transmigration, inhibiting the effects of cholesterol metabolism in addition to decreasing the progression of AS in ApoE^−/−^ mice (Wang et al., 2010b; Stumpf et al., 2013).

#### 5.2.5 Resveratrol

Resveratrol (RV), the most studied stilbene structure, is present in typical food sources such as grapes, berries, peanuts, and red wine, and, in some herbs, it is regarded as a strong antioxidant, among other properties (Sarubbo et al., 2017). In fact, research has identified several RV advantageous properties, making it possible for it to play crucial roles in the treatment of diseases including cancer, CVD, and AD, as well as other degenerative brain illnesses (Choi et al., 2012). Recent evidence suggests that the beneficial effects of RV may be related to altered epigenetic mechanisms. After being taken orally, RV can cause several chemical modifications, including oxidation, dehydroxylation, and demethylation, which can either directly inhibit the activity of epigenetic enzymes such as DNMTs, HDACs, or HATs or change the amount of substrate that is available for those enzymatic reactions (Griñán-Ferré et al., 2021). Since β-glucan-induced trained immunity depends on histone acetylation, under the action of the histone deacetylase activator RV (a sirtuin 1 activator), trained immunity is significantly inhibited and partially suppressed enhancement of IL-6 production (Cheng et al., 2014).

Regarding the effect on macrophages, RV can reverse the oxysterol-induced M2/M1 phenotypic switch (Buttari et al., 2014). RV regulates microglial M1/M2 polarization through PGC-1α under neuroinflammatory injury. Similar studies have shown that malibatol A (MA), an oligomer of RV, inhibits the expression of proinflammatory cytokines and M1 markers (CD16, CD32, and CD86) while increasing M2 in LPS-stimulated microglia markers (CD206 and YM-1) (Pan et al., 2015). RV has the atherosclerotic protective mechanism by regulating monocyte/macrophage differentiation, among other mechanisms, including inhibition of LDL oxidation, enhanced endothelial protection, reduction of trimethylamine N-oxide (TMAO) by gut flora, and inhibition of vascular smooth muscle cell (VSMC) proliferation and migration (Vasamsetti et al., 2016). RV suppressed LPS-induced RAW264.7 foam cell development by lowering ROS production and MCP1 expression through the Akt/Foxo3a and AMPK/Sirt1 pathways, which rely on NADPH oxidase 1 (Nox1) (Park et al., 2009; Dong et al., 2014a). According to clinical research, RV decreases the amount of TC and TG in individuals with dyslipidemia (Simental-Mendía and Guerrero-Romero, 2019).

#### 5.2.6 Salicylic acid

Radix *Salvia miltiorrhiza* (Danshen), which produces salvianolic acid B (SalB), has several medicinal actions, including antioxidant, anticancer, anti-inflammatory, and antiatherosclerotic qualities (Tang et al., 2022). Research showed that SalB induced the production of ABCA1 in differentiated THP-1 macrophages, which helped the HDL and ApoA-1-mediated cholesterol export. Additional mechanism experiments revealed that PPARγ and LXRα inhibitors might decrease the overexpression of ABCA1 triggered by SalB, which suggested that SalB promoted cholesterol efflux through a PPARγ/LXRα/ABCA1-dependent mechanism in THP-1 macrophages to minimize lipid buildup (Yue et al., 2015). Salvianolic acid B was discovered using a high-throughput screening experiment to be a powerful CD36 antagonist that prevents oxLDL absorption in RAW264.7 macrophages (Wang et al., 2010c).

### 5.3 Terpenoids

Terpenoids, also known as isoprenoids, are isoprene-based natural compounds having critical functions in every organism’s metabolism (Bergman et al., 2019). The terpenoid family of natural compounds, which includes several plant terpenoids, has been a valuable source of medicinal discoveries.

#### 5.3.1 Betulinic acid

Betulinic acid (BA), a natural pentacyclic triterpenoid, is an active compound in the bark of the birch tree *Betula* spp. (Betulaceae). BA has many biological effects, including anti-inflammatory, antiviral, antioxidant, and anticancer properties (Appiah et al., 2018). Research found that betulinic acid reduced atherosclerotic lesions, TG, TC, and LDL-C levels in ApoE^−/−^ mice by blocking the NF-κB signaling pathway and miR-33 expression (Zhao et al., 2013c). In RAW264.7 and THP-1 cells, betulin (a derivative of betulinic acid) consistently improved ABCA1/ABCG1-mediated cholesterol efflux by preventing the synthesis of SREBPs, which bound to E-box motifs in the ABCA1 promoter (Gui et al., 2016).

#### 5.3.2 Ginsenosides

The main active compounds in ginseng are called ginsenosides, which are triterpene glycosides of the dammarane type (Nah et al., 2007). Ginsenosides are expressed by the formula Rx, where x represents the separation from the thin-layer chromatography origin (Kim et al., 2017). The segments are labeled A for the most polar and H for the least polar (Kim et al., 2017). Protopanaxadiols, protopanaxatriols, and oleanane are the three main families of ginsenosides (ginsenoside Ro). Ginsenosides Rb1, Rb2, Rb3, Rc, Rd, Rg3, and Rh3 are examples of protopanaxadiols, which include sugar moieties on the C-3 position of dammarane-type triterpenes. Ginsenosides Re, Rf, Rg1, Rg2, and Rh1 are examples of the sugar moieties on the C-6 position dammarane-type triterpenes that make up protopanaxatriol (Lü et al., 2009; Kim et al., 2018). Ginseng is a well-liked supplement due to its wide range of pharmacological and therapeutic effects on aging, cancer, the cardiovascular system, diabetes, immune-regulatory function, and inflammation (Im, 2020). Ginsenoside Rg1 exerts positive effects on mesenchymal stem cells (MSCs). Ginsenoside Rg1 can influence HSC proliferation and migration, control HSCs/hematopoietic progenitor cell (HPC) differentiation, and slow down HSC aging. These findings may offer new approaches for increasing the homing rate of HSCs during HSC transplantation and for the treatment of graft-versus-host disease (GVHD) and other diseases caused by HSCs/HPC dysplasia (He and Yao, 2021).

Regarding the effect on macrophage polarization, ginsenoside Rg3 showed a positive effect on M2 polarization. After treatment with LPS, isolated mouse peritoneal macrophages significantly expressed several M1 marker genes, such as COX-2 (cyclooxygenase), iNOS, IL-1β, and TNFα. Pretreatment with Rg3 successfully restored a representative M2 marker (arginase-1), which was reduced after treatment with LPS (Koh et al., 2018). Ginsenoside Rg3 significantly reduced ox-LDL-induced atherosclerotic pathological changes in ApoE^−/−^ mice fed with HFD, upregulated PPARγ, and inhibited the activation of focal adhesion kinase (FAK) in the aorta, thus inhibiting the expression of VCAM-1 and ICAM-1 in the intima (Geng et al., 2020). Another crucial component of ginseng is ginsenoside Rd. Data from a RAW264.7 cell model showed that Rd inhibited the expression of the SR-A protein, followed by a decrease in the uptake of oxLDL and in the amount of cholesterol inside the cell (Li et al., 2011). An *in vivo* investigation revealed that Rd administration decreased the atherosclerotic plaque areas and oxLDL absorption in ApoE^−/−^ mice.

#### 5.3.3 Tanshinone IIA

Extracted from *Salvia miltiorrhiza* Bunge, tanshinone IIA (Tan IIA) is a significant lipophilic diterpene (Ono, 2018). Tan IIA can prevent or decrease the advancement of several illnesses, including cardiovascular diseases, cancers, cerebrovascular diseases, and Alzheimer’s disease, according to several experimental and clinical studies (Ding et al., 2020). One study demonstrated that TIIA treatment attenuated high glucose-induced kidney damage by modulating the DNA methylation of related genes Nmu, Fgl2, Glo, and Kcnip2 (Li et al., 2019). Tanshinone IIA consistently boosted ERK/Nrf2/HO-1 loop-mediated ABCA1- and ABCG1-mediated cholesterol efflux and decreased SR-A-mediated oxLDL absorption by inhibiting AP-1, which reduced cholesterol buildup in cells (Liu et al., 2014a). Another investigation using peritoneal macrophages from rats and macrophages generated from THP-1 revealed that tanshinone IIA treatment greatly raised ABCA1 mRNA and protein expression while considerably reducing CD36, suggesting simultaneous effects on cholesterol intake and efflux (Jia et al., 2016). In addition, a clinical trial has demonstrated that tanshinone IIA lowers hs-CRP in CAD patients (Li et al., 2017c).

#### 5.3.4 Ursolic acid

Ursolic acid (3B-hydroxy-12-urc-12-en-28-oic acid) is a pentacyclic triterpenoid produced from plants that have antioxidant, anti-inflammatory, and neuroprotective properties (Rong et al., 2022). A study on skin cancer reported that ursolic acid therapy reduces hypermethylated CpG islands of the Nrf2 gene promoter region in mouse epidermal cells, restoring Nrf2 expression, accomplished by lowering the production of epigenetic-modifying enzymes such as DNA methyltransferases (Kim et al., 2016). A recent study found that ursolic acid improved the transport of cholesterol from LDL-loaded macrophages to ApoA-1 through autophagy without changing the levels of ABCA1 and ABCG1 mRNA or protein in MPMs (Leng et al., 2016). *In vivo*, UA therapy dramatically decreased the size of the atherosclerotic lesion and increased macrophage autophagy in LDLR^−/−^ mice (Leng et al., 2016).

#### 5.3.5 Zerumbone

A naturally occurring substance called zerumbone is derived from pinecone or shampoo ginger, *Zingiber zerumbet* L. Smith, and contains several pharmacological properties, including antiulcer, antioxidant, anticancer, and antibacterial (Rosa et al., 2019). Earlier research found that zerumbone significantly reduced the inflammatory response caused by LPS in *in vitro* and *ex vivo* trials using the macrophages employed in this investigation by inhibiting the activation of the ERK-MAPK and NF-κB signaling pathways and the NLRP3 inflammasome (Su et al., 2021). Studies in a rabbit model fed cholesterol show that zerumbone can stop the development of atherosclerotic lesions (Hemn et al., 2015). Zerumbone reduced the expression of SR-A and CD36 mRNA in vitro studies by controlling AP-1 and NF-κB suppression, which blocked the absorption of acLDL by THP-1 macrophages (Eguchi et al., 2007). Additionally, zerumbone treatment of THP-1 macrophages resulted in a considerable decrease in cholesterol levels through increasing the mRNA and protein levels of ABCA1, but not ABCG1, and ERK1/2 phosphorylation (Zhu and Liu, 2015).

### 5.4 Carotenoids

Carotenoids represent a class of pigmented terpenoids. The human diet contains around 50 of more than 700 carotenoids identified in nature, with about half present in human blood and tissues (Krinsky and Johnson, 2005). Lycopene, lutein, zeaxanthin, β-cryptoxanthin, α-carotene, and β-carotene are the main carotenoids in human serum (Krinsky and Johnson, 2005). According to epidemiologic research, it may be linked to better cognitive and visual abilities and a lower chance of developing chronic conditions, including cancer, CVD, and age-related macular degeneration (AMD) (Moran et al., 2018).

#### 5.4.1 Astaxanthin

A natural xanthophyll carotenoid called astaxanthin (3,3′-dihydroxy-β,β′-carotene-4,4′-dione) is present in various marine species, such as *Haematococcus pluvialis*, *Chlorella zofingiensis*, *Chlorococcum*, and *Phaffia rhodozyma* (Hussein et al., 2006). It has been suggested that it has anti-inflammatory, antioxidant, and neuroprotective properties, and research from different experimental models has demonstrated that these properties are linked to a decreased expression of proinflammatory cytokines and a decreased production of ROS and free radicals (Kim et al., 2020). Xue et al. (2017) reported that astaxanthin prevents oxidative stress and apoptosis, which reduces the hematopoietic damage caused by whole-body radiation in mice. Yang et al. (2017) stated that astaxanthin could demethylate certain promoters of particular genes, which may help increase the stability of the total chromatin structure. Only at high dosages does astaxanthin enhance the expression of ABCA1/G1 (up to 2.0- and 3.2-fold at the protein level), which promotes ApoA-1/HDL-mediated cholesterol efflux (Iizuka et al., 2012).

#### 5.4.2 β-Carotene

β-Carotene (BC), a precursor to vitamin A, is present in a greater variety of fruits and vegetables. It is frequently used in foods as an antioxidant and natural colorant (Zhao et al., 2020). Lower overall, CVD, heart disease, stroke, cancer, and other causes of death are linked to a higher β-carotene biochemical state (Huang et al., 2018). Kim et al. (2019) suggested that BC can regulate epigenetic modifications for its anticancer effects in colon cancer stem cells. Furthermore, endogenous β-carotene 15,15′-monooxygenase 1 may convert 9-cis-c into 9-cis retinoic acid or other retinoids, activate the retinoid X receptor (RXR), and stop foam cell formation and the development of AS (Zolberg Relevy et al., 2015).

#### 5.4.3 Lycopene

Lycopene, a member of the carotenoid family, is mostly found in foods such as tomatoes (particularly the red kind), watermelons, and red pomelo (Zhan et al., 2021). Lycopene is widely recognized for its anti-inflammatory and antioxidant properties and ability to affect important bodily metabolic processes (Han et al., 2016). Napolitano et al. (2007) demonstrated that lycopene reduced cholesterol buildup by upregulating IL-10 secretion in human peripheral blood monocyte-derived macrophages (HMDMs) and THP-1 macrophages and downregulating SR-A mRNA expression and lipid synthesis. Furthermore, HMG-CoA reductase inhibition, RhoA inactivation, an increase in PPARγ and LXRα activation, and ultimately an improvement in ABCA1 and caveolin 1 expression may all contribute to the potential cascade impact of lycopene in lowering foam cell formation (Palozza et al., 2011).

#### 5.4.4 Retinoids

Retinol, generally known as vitamin A1, and its natural derivatives, 9-cis retinoic acid (9-cis-RA) and all-trans retinoic acid (ATRA), are thought to be prospective therapeutic agents for the prevention of AS development because they can promote macrophage cholesterol efflux. Retinoids reportedly cause epigenetic alterations that cause stem cell differentiation (Gudas, 2013). Through binding to the RARs, ATRA modifies how the retinoic acid receptors (RARs) interact with various protein elements of the transcription complex at multiple genes in stem cells. The epigenetic marks on histones or DNA are added to or removed by some of these protein components of the transcription complex, altering the chromatin structure and leading to the departure from the self-renewing, pluripotent stem cell state (Gudas, 2013). According to research, 9-cis-RA and ATRA can significantly increase the expression of ABCA1, ABCG1, and ApoE in THP-1 macrophages and the efflux of cholesterol to ApoA-1 in RAW264.7 macrophages (Langmann et al., 2005). Additionally, 9-cis-RA has been connected to the ABCA1-mediated cholesterol efflux from J774 macrophages, THP-1-derived macrophages, and RAW264.7 macrophages (Schwartz et al., 2000; Kiss et al., 2005).

### 5.5 Phenylpropanoids

#### 5.5.1 Ferulic acid

Ferulic acid (FA) ((E)-3-(4-hydroxy-3-methoxy-phenyl) prop-2-enoic acid), a caffeic acid derivative, can be isolated from several Chinese herbal medicines, including *Cimicifuga racemosa*, *Angelica sinensis*, and Rhizoma Ligustici Chuanxiong, as well as from plants that are commonly found in our diet, including *Oryza sativa*, *Glycine max*, and *Zea mays* (Li et al., 2022). FA has free radical scavenging and antioxidant properties that have a wide range of potential applications in the prevention and treatment of CVD and the management of cancer, as well as hepatoprotective, antimicrobial, and anti-inflammatory therapies (Li et al., 2022). FA, a metabolite of chlorogenic acid, has an improving effect on HDL-mediated cholesterol efflux from macrophages by increasing the expression of ABCG1 and SR-BI (Uto-Kondo et al., 2010).

#### 5.5.2 Chlorogenic acid

Chlorogenic acid (CA) is a phenolic molecule from the hydroxycinnamic family found in drinks made from herbs, fruits, and vegetables. It is recognized for its antioxidant capabilities against free radicals (Santana-Gálvez et al., 2017). In ApoE^−/−^ mice fed a diet high in cholesterol, CA decreased the percentage and total atherosclerotic lesion area, as well as the aortic dilatation and serum levels of TC, LDL-C, and TG (Wu et al., 2014). Through the upregulation of the transcription of PPARγ, LXRα, ABCA1, and ABCG1 in *in vitro* mechanistic studies, CA repressed foam cell growth and decreased the oxLDL-induced neutral lipid and cholesterol accumulation in RAW264.7 macrophages (Wu et al., 2014). In HepG2 cells, chlorogenic acid enhanced mRNA expression of ABCA1, CYP7A1, and AMPKα2 and facilitated the efflux of TC and triacylglycerol (Hao et al., 2016).

#### 5.5.3 Lignans

The largest concentration of lignans, which are bioactive, non-nutritive, non-caloric phenolic plant chemicals, is found in flax and sesame seeds (Peterson et al., 2010). Dietary lignans demonstrate strong antiviral, antioxidant, anticancer, and antiatherosclerotic properties *via* functioning as phytoestrogens (Peterson et al., 2010).

##### 5.5.3.1 Arctigenin

Across hundreds of years, people all over the world have used the roots of *Arctium lappa*, also known as larger burdock, as food and traditional herbal medicine. The seeds of the *Arctium lappa* plant contain arctigenin, a phenylpropanoid dibenzylbutyrolactone lignin (Nam and Nam, 2020). The antibacterial, antiviral, antioxidant, anti-inflammatory, and anticancer properties of arctigenin have been demonstrated (Maxwell et al., 2018). Arctigenin boosted the expression of ApoE, ABCA1, and ABCG1 in oxLDL-loaded THP-1 macrophages, increasing cholesterol efflux (Xu et al., 2013).

##### 5.5.3.2 Honokiol

Honokiol (HKL) [2-(4-hydroxy-3-prop-2-enyl-phenyl)-4-prop-2-enyl-phenol] is a naturally occurring biphenolic chemical with a low molecular weight, which is obtained from the bark of magnolia trees and is utilized in traditional Chinese medicine (Fried and Arbiser, 2009). It possesses analgesic, anti-inflammatory, antioxidant, anti-tumor, and neuroprotective activities as a pharmaceutical (Pillai et al., 2015). Honokiol could activate the RXR/LXR heterodimer, inducing the ABCA1 expression and improving cholesterol efflux from MPMs (Kotani et al., 2010). According to another study, honokiol boosted ABCA1 expression by interacting with RXRβ. Additionally, it boosted the expression of ABCG1 and ApoE (Jung et al., 2010).

##### 5.5.3.3 Sesamin

Sesamin, a naturally occurring lignin compound, is isolated from sesame seeds and has many positive health effects, including anti-inflammatory, anticancer, anti-hypertension, anti-thrombotic, antidiabetic, anti-atherogenic, anti-obesity, and lipolytic effects (Dalibalta et al., 2020). It also can reduce damage to the intestine, kidneys, heart, brain, and liver (Wang et al., 2021b). Studies have shown that sesamin inhibits LPS-induced macrophage-derived chemokine expression through ER, PPAR-α, MAPK-p38 pathway, NFκB-p65 pathway, and epigenetic regulation (Hsieh et al., 2014). Sesamin boosted cholesterol efflux from RAW264.7 macrophages and decreased the oxLDL-induced accumulation of cholesterol, most likely by activating PPARγ, LXRα, and ABCG1 (Liu et al., 2014b). Sesamin reduced AS in ApoE^−/−^ mice by stifling vascular inflammation, according to *in vivo* research (Wu et al., 2010).

### 5.6 Alkaloids

#### 5.6.1 Berberine

Berberine (BBR) is a naturally occurring substance extracted from herbs, including *Coptis chinensis* and *Berberis vulgaris*. BBR has been identified as a safe and effective treatment for type 2 diabetes and hyperlipidemia with new mechanisms since 2004 (Kong et al., 2004). Over the past 10 years, several studies have demonstrated the clinical effectiveness of BBR in decreasing lipids and glucose (Wang et al., 2021c). The investigation results demonstrated that BBR administration mostly impacted enzymes involved in histone acetylation and methylation (Zhang et al., 2016). The expression of the proteins H3K4me3, H3K27me3, and H3K36me3 reduced after BBR administration, according to Western blotting tests conducted concurrently (Zhang et al., 2016). By encouraging LXRα/ABCA1-dependent cholesterol efflux, BBR reduced the development of foam cells in THP-1 macrophages (Lee et al., 2010). Nevertheless, ABCG1, SR-BI, CD36, and SR-A were unaffected by berberine. The impact of BBR on macrophages is also mediated by other pathways, including AMPK/Sirt1 activation, autophagy induction, and adipocyte enhancer-binding protein 1 suppression (Huang et al., 2012; Chi et al., 2014; Kou et al., 2017). Moreover, in a rat model of adjuvant arthritis, BBR treatment restrained the phagocytic function of macrophages and restored the balance of M1/M2 by reducing the levels of M1 cytokines (TNF-α, IL-1β, and IL-6), increasing the levels of M2 cytokines (IL-10 and TGF-β1), increasing the expression of arginase 1(Arg1) (M2 marker), and decreasing the expression of iNOS (M1 marker) (Zhou et al., 2019).

#### 5.6.2 Piperine

Long and black peppers contain piperine (Srinivasan, 2007). Previous research has demonstrated that piperine has a variety of pharmacological properties. In terms of pharmaceuticals, piperine decreases depressive disorders, prevents hepatotoxicity, and reduces obesity and diabetes (Nogara et al., 2016). According to studies, piperine suppresses the development of adipocytes by dynamically controlling histone modifications and regulating the expression of genes involved in adipogenesis and lipolysis (Park et al., 2019). In THP-1-differentiated human macrophages, piperine was likewise observed to increase ABCA1 protein expression. However, it did not affect ABCG1 or SR-BI expression (Wang et al., 2017a).

#### 5.6.3 Rutaecarpine

Rutaecarpine (8,13-dihydro-7H-indolo-[2′,3′:3.4]-pyrido [2,1-b]-quinazolin-5-one) is an alkaloid first isolated from *E. rutaecarpa*. Earlier studies demonstrated that rutaecarpine possesses many biological and pharmacological features, including the ability to cause diuresis, sweating, uterotonic action, brain function improvement, antinociception, and anti-obesity (Jayakumar et al., 2021). Studies showed that rutaecarpine increased cholesterol efflux by upregulating the expression of ABCA1 and SR-BI *in vitro* (RAW264.7 macrophages and HepG2 cells) and *in vivo* (ApoE^−/−^ mice) (without changing ABCG1 and CD36) (Xu et al., 2014). This reduced the lipid buildup and foam cell formation. Through this method, rutaecarpine decreased the growth of atherosclerotic plaque in ApoE^−/−^ mice (Xu et al., 2014).

### 5.7 Others

#### 5.7.1 Astragalus polysaccharides

The primary active ingredient of *Astragalus membranaceus*, *Astragalus* polysaccharides (APS), is widely used in clinical applications as an immunomodulator (Li et al., 2012). It has several bio-activities, including anti-inflammatory, proliferative, and immune-regulating effects, and a molecular weight of 3.6 × 10^4^ Da (Sun et al., 2021a). Studies have demonstrated that APS significantly abrogates LPS-induced IL-6 levels in THP-1 macrophages (Long et al., 2022). ABCA1 expression in foam cells exposed to TNF-α increases in response to APS (Wang et al., 2010a). As a result, APS increases the outflow of cholesterol and reduces fat accumulation. According to further research, TNF-α-induced NF-κB activation in foam cells generated from THP-1 was reversed by APS (Wang et al., 2010a).

#### 5.7.2 Diosgenin

Diosgenin has gained more attention recently due to its efficacy in treating several metabolic diseases, including diabetes, CVD, neurological conditions, osteoporosis, and hyperlipidemia, as well as its anticancer effects, which are mediated *via* multiple targets and regulate a variety of signals (Sun et al., 2021b). By preventing the nuclear translocation of the Notch intracellular domain in THP-1 cells, diosgenin prevented AS (Binesh et al., 2018). By preventing the induction of ICAM1, VCAM1, and endothelial lipase, it also prevented the adherence of TNF-α-induced leukocytes to activated endothelium cells (Wu et al., 2015b). Diosgenin is a naturally occurring compound capable of modulating M1 polarization (Saqib et al., 2018). Additionally, dioscin prevented systemic inflammation and the LOX-1/NF-κB pathway in MPMs from rats with atherosclerotic arteries, inhibiting the absorption of oxLDL (Wang et al., 2017b).

#### 5.7.3 *Panax notoginseng* saponins

The primary bioactive components of *Panax notoginseng* (*P. notoginseng*) are known as *Panax notoginseng* saponins (PNS), which include several saponins of the dammarane type (Xu et al., 2019). PNS have several cardiovascular preventive properties, including avoiding endothelial dysfunction, boosting blood flow, inhibiting the production of foam cells, antioxidation, anti-inflammation, and antithrombosis (Yuan et al., 2011). According to Duan et al. (2022), PNS regulate the miR-194 promoter, miR-194, and MAPK methylation using cellular assays and blinded, controlled trials. PNS increased ABCA1 expression in macrophages, which reduced the buildup of cholesterol esters (Jia et al., 2010). PNS, at a dosage of 100 mg/kg per day, reduced foam cell development in rats with AS caused by zymosan A, according to *in vivo* research (Yuan et al., 2011).

#### 5.7.4 Emodin

Emodin is an anthraquinone derivative isolated from *Polygonum multiflorum* (Ma et al., 2015). It has various therapeutic actions, including anti-tumor, anti-inflammatory, antioxidant, and anti-virus properties (Zheng et al., 2019). Studies have shown that emodin can bidirectionally regulate macrophage polarization and epigenetic regulation of macrophage memory (Iwanowycz et al., 2016). Emodin prevented H3K27 trimethylation (H3K27m3) marks from being removed from, and H3K27ac marks from being added to, genes needed for M1 or M2 polarization of macrophages (Iwanowycz et al., 2016). By activating the PPARγ/LXRα/ABCA1 signaling pathway, emodin boosted ApoA-1-mediated cholesterol efflux from THP-1 macrophages. Emodin also reduced diet-induced AS in rabbits (Hei et al., 2006; Fu et al., 2014).

## 6 Summary and perspectives

Evidence suggests that low-grade inflammation, predominantly driven by the immune system, plays a critical role in the development of AS (Leentjens et al., 2018). Although anti-inflammatory medications, such as canakinumab and colchicine, have been recently proven to lower the risk of CVD, there are still significant side effects and a high residual risk (Ridker et al., 2017; Tardif et al., 2019). Therefore, innovative therapies are urgently needed, and trained immunity provides interesting new pharmacological targets for new drug therapies. With enhanced production of pro-atherosclerotic cytokines/chemokines and higher foam cell generation, trained monocytes and macrophages showed a strong pro-atherosclerotic character. This is accomplished by epigenetic reprogramming of histone methylation levels and metabolic rewiring. These processes occur not only in circulating monocytes but also in myeloid progenitor cells, which ensure a long-term state of hyperactivation of innate immune cells. This review describes the aforementioned mechanisms in detail.

Although trained immunity is an immunological memory that is not disease-specific, different trained immune programs have different levels of disease specificity (Mulder et al., 2019). Natural products serve as a desirable resource in the search for novel therapies due to their high structural variety and biodiversity. Many natural products have been potential candidates for regulating immune training through different mechanisms, such as RV and EGCG. This paper provides an overview of anti-ASCVD natural products, such as flavonoids, phenols, terpenoids, carotenoids, phenylpropanoids, and alkaloids, that potentially modulate trained immunity. Although *in vivo* studies of AS models already exist for these natural compounds, making these compounds more promising, there is currently less evidence that these natural compounds can directly modulate training immunity. Further studies are needed to reveal possible pathways by which natural products act on trained immunity.

## Data Availability

The original contributions presented in the study are included in the article/Supplementary Material, further inquiries can be directed to the corresponding author.

## Glossary

ASCVD: atherosclerotic cardiovascular diseases
AS: atherosclerosis
ABCA1: ATP-binding cassette transporter A1
APS: *Astragalus* polysaccharides
ATRA: all-trans retinoic acid
AMD: age-related macular degeneration
ABCG1: ATP-binding cassette transporter G1
ATP: adenosine triphosphate
AMPK: AMP-activated protein kinase
AP-1: activating protein-1
BCG: bacille Calmette–Guérin
BA: betulinic acid
BC: β-carotene
BBR: berberine
CA: chlorogenic acid
CD36: cluster of differentiation 36
CAD: coronary artery disease
CoA: acetyl coenzyme A
CCL2: chemokine C–C motif ligand 2
CVD: cardiovascular diseases
C3G: Cyanidin-3-O-glucoside
chrysin: 5,7-dihydroxyflavone
CARM1: coactivator-associated arginine methyltransferase 1
ceRNA: competing endogenous RNA
DAMP: damage-associated molecular pattern
DNMTs: DNA methyltransferases
EGCG: epigallocatechin-3-gallate
EA: ellagic acid
FAO: fatty acid oxidation
FAS: fatty acid synthase
FAK: focal adhesion kinase
GM-CSF: granulocyte–macrophage colony-stimulating factor
GS: glutamine synthetase
GVHD: graft-versus-host disease
HATi: histone acetyltransferase inhibitor
HATs: histone acetyltransferases
HDACs: histone deacetylases
HDL-C: HDL-cholesterol
HFD: high-fat diet
HDL: High-density lipoprotein
HSCs: hematopoietic stem cells
HMG-CoA: 3-hydroxy-3-methylglutaryl-coenzyme A
H3K4me3: histone H3 lysine 4 trimethylation
HIF-1α: hyperoxia-inducible substance 1α
HK-II: hexokinase II
H3K27ac: histone 3 lysine 27 acetylation
H3K4me1: histone 3 lysine 4 methylation
hesperidin: 3′,5,7,-trihydroxy-4′-methoxyflavanone
H3K4me2/3: H3K4 di- and tri-methylation
HDAC1–2: histone deacetylase 1–2
HPC: hematopoietic progenitor cell
HMDMs: human peripheral blood monocyte-derived macrophages
H3K27m3: H3K27 trimethylation
iNOS
inducible nitric oxide synthase
IL: interleukin
ICAM-1: intercellular adhesion molecule 1
JNK: c-Jun N-terminal kinases
LDL: low-density lipoprotein
LDs: lipid droplets
LXR: liver X receptor
LPS: lipopolysaccharide
lncRNAs: long non-coding RNAs
LDL-C: LDL-cholesterol
MCP-1: monocyte chemoattractant protein-1
MMP-2: matrix metalloproteinases 2
m5C: methylate cytosine
mTOR: mammalian target of rapamycin
MSCs: mesenchymal stem cells
NLRP3
NOD-, LRR-, and pyrin domain-containing protein 3
NO: nitric oxide
Nox1: NADPH oxidase 1
oxLDL: oxidized LDL
OXPHOS: oxidative phosphorylation
oxPAPC: oxidized phospholipids made of 1-palmitoyl-2-arachidonoyl-sn-glycero-3-phosphorylcholine
PPARγ: peroxisome proliferator-activated receptor γ
PPP: pentose phosphate pathway
PFKFB3: 6-phosphofructo-2-kinase/fructo-2, 6-bisphosphatase
PDK1: pyruvate dehydrogenase kinase isozyme 1
PI3K: phosphoinositide 3-kinase
PON-1: paraoxonase-1
PEA: pomegranate ellagic acid
PCSK9: proprotein convertase subtilisin/kexin type 9
PCA: protocatechuic acid
PNS: *Panax notoginseng* saponins
quercetin
3,3',4',5,7-pentahydroxyflavone
RXR: retinoid X receptor
ROS: reactive oxygen species
Runx1: Runt-related transcription factor 1
RCT: reverse cholesterol transport
RV: resveratrol
RARs: retinoic acid receptors
SalB: salvianolic acid B
SAM: S-adenosylmethionine
SR-A: type A scavenger receptor
SR-BI: scavenger receptor class B type I
SDH: succinate dehydrogenase
STK11: serine/threonine kinase 11
TNF-α: tumor necrosis factor-α
TCA cycle: tricarboxylic acid cycle
TLR4: Toll-like receptor 4
TC: total cholesterol
TMAO: trimethylamine N-oxide
Tan IIA: tanshinone IIA
ursolic acid
3B-hydroxy-12-urc-12-en-28-oic acid
9-cis-RA
9-cis retinoic acid
VCAM-1: vascular cell adhesion molecule 1
VSMCs: vascular smooth muscle cells
WTD: Western-style diet
WDR5: WD repeat-containing protein 5
2-DG
2-deoxy-d-glucose
2-DG6P
2-deoxy-d-glucose-6-phosphate.
