# Supplementary material for: Trained immunity in monocyte/macrophage: Novel mechanism of phytochemicals in the treatment of atherosclerotic cardiovascular disease
Source: Front Pharmacol. 2023 Feb 21;14:1109576. doi: 10.3389/fphar.2023.1109576 (PMC9989041; doi:10.3389/fphar.2023.1109576)
Supplement: Supplementary file 1 [file Table2.docx]

**Table 2: Anti-atherosclerotic natural product that may target trained immunity**

| **Phytochemical agent** | **Experimental model** | **Dose, route, and duration of administration** | **Finding** | **Mechanism and effect** | **Potential Trained Immunity Relevance** | **Bioavailability** | **Ref** |
| --- | --- | --- | --- | --- | --- | --- | --- |
| **Flavonoids** | | | | | | | |
| Alpinetin  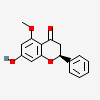 | THP-1 macrophages and HMDMs | 50, 100, and 150 µg/mL for 24 H. | ↓lipid accumulation; ↓foam cell formation | Regulating PPARγ/LXRα/ABCA1/ABCG1 pathway | NLRP3 and HIF-1α inhibitor | The absolute bioavailability is 15.10 ± 5.72% | ^136-138^ |
|  | THP-1 macrophages | 50, 100, and 200 µg/mL for 1 H. | ↓inflammation | Inhibiting TLR4/NF-κB/ MAPK pathway |  |  |  |
| Anthocyanins  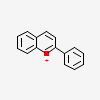 | male ApoE^−/−^ mice | 300 mg/(kg·d), p.o.;for 20 weeks | ↓lipid accumulation; ↓inflammation | Decreasing serum triglyceride, total cholesterol, and non-HDL cholesterol | NLRP3 and ROS inhibitor | Poor; only trace concentrations in plasma | ^139 140^ |
| Cyanidin-3-O-β-glucoside | male Sprague-Dawley rat | 150 mg/kg, p.o.;for 4 weeks | ↓lipid accumulation; | Decreasing serum levels of total cholesterol, free cholesterol, triglycerides, and free fatty acids | Histone methylation regulators |  | ^141^ |
| Baicalin  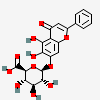 | male New Zealand rabbits | 224 mmol/kg, p.o.;for 8 weeks | ↓lipid accumulation; ↓foam cell formation | Regulating PPARγ/LXRα/ABCA1/ABCG1 pathway | Epigenetic regulator | The absolute bioavailability is only 2.2% after oral administration | ^142 143^ |
| Chrysin  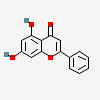 | RAW264.7 macrophages | 10 µM for 24 H. | ↓lipid accumulation; ↓foam cell formation | Regulating PPARγ/LXRα/ABCA1/ABCG1 pathway; down-regulation of SR-A1 and SR-A2 | NLRP3 and IL-1β inhibitor | The absolute bioavailability is only 1% after oral administration | ^144 145^ |
| Daidzein  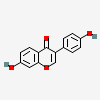 | male Wistar rats | 10 and 30 mg/kg p.o.;for 3 weeks | ↓lipid accumulation; | Decreasing the serum cholesterol and increasing TG level | Epigenetic regulator | Poor | ^146 147^ |
| Ellagic acid  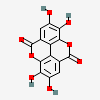 | J774A1 murine macrophages | 1 and 5 µM for 24 H. | ↓lipid accumulation | Regulating PPARγ/LXRα/ABCA1/ABCG1 pathway | Histone methylation regulators | Hardly exceeds 100 nM in human plasma. | ^148-150^ |
|  | hamster model with HFD | 177 mg/(kg·bw)，p.o.;for 4 weeks | ↓lipid accumulation | Enhancing fecal bile acid; up-regulation of PPARγ/LXRα/ABCA1/ABCG1 pathway |  |  |  |
| Epigallocatechin gallate  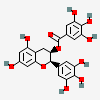 | THP-1 macrophages | 40, and 80 µg/mL for 16 H | ↓lipid accumulation; ↓inflammation | Inhibiting Nrf2/Keap1/ NF-κB pathway | Histone acetyltransferase inhibitor | The absolute bioavailability is only 4.95% after oral administration | ^151-154^ |
|  | male LDLr^−/−^ mice | 25 mg/(kg·bw) for 4 weeks;  (EGCG-loaded nanoparticles) | ↓lesion surface areas of aortic arches | Decreasing inflammatory factors in mouse peritoneal macrophages |  |  |  |
|  | THP-1 macrophages | 10 µM for 24 H. | ↓lipid accumulation; ↓foam cell formation | Blocking the oxLDL-induced upregulation of SR-A |  |  |  |
| Hesperidin  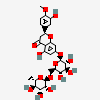 | THP-1 macrophages | 5, 10 and 15 µM for 24 H. | ↓foam cell formation | Regulating PPARγ/LXRα/ABCA1 pathway | Histone acetylation regulators | About 20% | ^155 156^ |
| Icarin  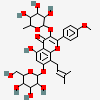 | male Wistar rats | 30 and 60 mg/kg p.o.;for 4 weeks | ↓lipid accumulation; ↓inflammation; ↓oxidative stress | Downregulation of p-p38 MAPK | - | About 12% | ^157-159^ |
|  | male apoE^−/−^ mice, RAW264.7 macrophages | 30 and 60 mg/kg p.o.;for 10 weeks; 5 and 10 µM for 1 H. | ↓lesion area; ↓macrophage infiltration | Inhibiting CX3CR1-CX3CL1 interaction |  |  |  |
| Pratensein  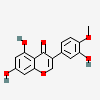 | HepG2 human hepatoma cells | 2.08 µM for 18 H. | ↓lipid accumulation | Upregulating the CLA-1 expression | - | - | ^160^ |
| Puerarin  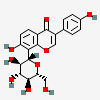 | THP-1 macrophages | 25, 50, and 100 µg/mL for 24 H. | ↓lipid accumulation; ↓foam cell formation | Regulating miRNA-7/ STK11 and AMPK/PPARγ/LXRα/ABCA1 pathway | Epigenetic regulator | The absolute bioavailability is only 7% after oral administration | ^161-163^ |
|  | male white rabbits | 0.1, 0.2, and 0.4 g/kg p.o.;for 90 days | ↓lesion area; ↓migration and reproduction of VSMCs | Decreasing PCNA and PDGF-A expressions |  |  |  |
| Quercetin  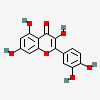 | THP-1 macrophages | 50, 100, and 200 µg/mL for 24 H. | ↓lipid accumulation | Regulating PPARγ/LXRα/ABCA1 pathway | Demethylation | The absolute bioavailability is only 1% after oral administration | ^164-166^ |
|  | male apoE^−/−^ mice | 12.5 mg/kg p.o.;for 8 weeks | ↑RCT | Increasing expression levels of ABCA1 and ABCG1 |  |  |  |
| Silymarin  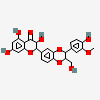 | THP-1 macrophages | 5, 10, 20 and 30 µM for 24 H. | ↑RCT | Promoting PPARγ/ ABCA1 pathway | Histone deacetylase inhibitor | About 23-47% | ^167-170^ |
|  | male Wistar rats | 300 and 600 mg/kg p.o.;for 60 days | ↓lipid accumulation | Decreased both serum and hepatic total cholesterol, triglycerides, VLDL-C, LDL-C and increasing HDL-C |  |  |  |
| **Phenols** | | | | | | | |
| Curcumin  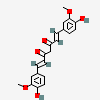 | male apoE^−/−^ and SR-A^−/−^ mice, J774.A1 macrophages | 20 mg/kg p.o.;for 4 weeks; 5, 10, 20 and 40 µM for 24H. | ↓lipid accumulation; ↓foam cell formation | Decreasing the SR-A-dependent oxLDL uptake and increasing the ABCA1-dependent cholesterol efflux; Activating AMPK-SIRT1-LXRα pathway; Activating Nrf2/HO-1 pathway; Inhibition of the p38MAPK pathway | NLRP3 inhibitor and Epigenetic regulator | The absolute bioavailability is less than 1 % after oral administration | ^171-176^ |
| Paeonol  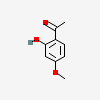 | male apoE^−/−^ mice, J774.A1 macrophages | 100 mg/kg p.o.;for 4 weeks; 25, 50 and 100 µM for 10H. | ↓lipid accumulation; ↓foam cell formation | Activating LXRα/ABCA1 pathway | Mediating the AKT/mTOR signaling pathway | Less than 10% | ^177-181^ |
|  | male apoE^−/−^ mice, RAW264.7 macrophages | 150 mg/kg p.o.;for 8 weeks; 5, 10 and 50 µM for 24H. | ↓lipid accumulation; ↓foam cell formation | Activating ABCA1 |  |  |  |
|  | VECs isolated from the rat thoracic aorta | 15, 30, 60, 120, 240 and 480 µM for 12, 24 AND 48H. | ↓monocyte adhesion; ↓inflammation;↑endothelial protection | Inhibiting PI3K/Akt/NF-κB pathway; promotion of miR‑126 expression |  |  |  |
| Polydatin  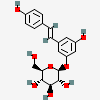 | Peritoneal macrophages of ApoE^−/−^ mice | 8.9 µg/mL for 24 and 48 H. | ↓foam cell formation; ↓inflammation | Regulating PPARγ signaling pathways | - | - | ^182^ |
| Protocatechuic acid  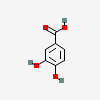 | male apoE^−/−^ mice, MAECs and MPMs | 25 and 30 mg/kg p.o.;for 1 weeks; 5, 10 and 50 µM for 24H. | ↑endothelial-dependent vasodilation | Improving vascular tetrahydrobiopterin levels | - | - | ^183 184^ |
| Resveratrol  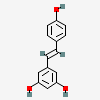 | male apoE^−/−^ mice, THP-1 macrophages | 10 mg/kg p.o.;for 6 weeks; 25-100 µM for 2H. | ↓monocyte differentiation;↓inflammation | Activating AMPK-α | Histone deacetylase activator and inhibit β-glucan-induced trained immunity | The absolute bioavailability is almost 0 after oral administration | ^119 185-187^ |
|  | THP-1 macrophages | 2.5 µM for 6H. | ↓lipid accumulation; ↓foam cell formation | Regulating the expression of MCP-1 and activating the AMPK/SIRT1/ PPARγ signaling pathway |  |  |  |
| Salicylic acid  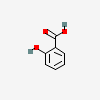 | AMPK β1-deficient BMDMs | 3 mM for 30H. | ↓lipid accumulation; ↓foam cell formation | Activating AMPK-β | Regulating BMDMs | - | ^188^ |
| **Terpenoids** | | | | | | | |
| Betulinic Acid  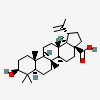 | male apoE^−/−^ mice, THP-1 macrophages | 50 mg/kg p.o.;for 8 weeks; 1 µg/ml for 12, 24 and 48H. | ↓inflammation; ↓lesion area; | Blocking NF-Κb/miRNA-33s/ABCA1 pathway | - | Poor; bescause of extreme low aqueous solubility | ^189 190^ |
|  | male apoE^−/−^ mice, RAW264.7 macrophages | 20 and 40 mg/kg p.o.;for 12 weeks; 1 µg/ml for 12, 24 and 48H. | ↓lipid accumulation; ↑fecal cholesterol efflux | Suppressing the expression of SREBPs |  |  |  |
| Ginsenosides  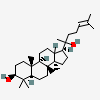 | male apoE^−/−^ mice, mouse primary peritoneal macrophages | 50 mg/kg i.p.;for 8 weeks; 10, 20, 40 and 80 µM for 24H. | ↓lipid accumulation; ↑AS plaque stability | Ginsenoside Rb1 increasing AMPK phosphorylation | Ginsenoside Rg1 affecting HSC | Ginsenoside Rb1 has an extremely low oral bioavailability of 0.28–1.18% | ^191-193^ |
|  | male apoE^−/−^ mice, HUVECs | 15 and 30 mg/kg p.o.;for 4 weeks; 7.5, 15 and 30 µM for 48H. | ↑protecting endothelial cells; ↓AS | Regulating the ICAM-1, VCAM-1 expression in HUVECs and vascular endothelium via PPARγ/FAK pathway |  | Poor | ^194 195^ |
| Tanshinone IIA  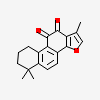 | male apoE^−/−^ mice, mouse peritoneal macrophages | 10, 30 and 90 mg/kg p.o.;for 20 weeks; 0.1, 1 and 10 µM for 48H. | ↓lesion area; ↓oxidative stress | PPARγ antagonism | Demethylation | The absolute bioavailability is less than 3.5% after oral administration | ^196-199^ |
|  | male apoE^−/−^ mice, THP-1 macrophages | 30 mg/kg p.o.;for 12 weeks; 10 µM for 24H. | ↓lesion area; ↓lipid accumulation | Inhibition of AP-1; Regulating ERK/Nrf2/HO-1 pathway |  |  |  |
| Ursolic acid  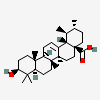 | female LDLR^−/−^ mice; RAW264.7 macrophages | 50 mg/kg p.o.;for 11 weeks; 10 µM for 6H. | ↑cholesterol efflux; ↓lesion area | Promoting autophagy and suppressing IL-1β secretion | Epigenetic regulator | Poor | ^200-202^ |
| Zerumbone  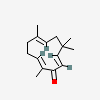 | THP-1 macrophages | 5, 10 and 20 µM for 0.5 H. | ↓DiI-acLDL uptake | Suppressing the SR-A and CD36 mRNA expression via regulating AP-1 and NF-κB repression | NLRP3 inhibitor | Poor | ^203-207^ |
|  | THP-1 macrophages | 10, 30, 60 and 100 µM for 24H. | ↑cholesterol efflux | Upregulation of mRNA and protein levels of ABCA1 |  |  |  |
|  | New Zealand white rabbit | 8, 16 and 20 mg/kg p.o.;for 4 weeks | ↓lesion area; ↓lipid accumulation; ↓oxidative stress | Suppressing lipid peroxidation and oxidative stress damage |  |  |  |
| **Carotenoids** | | | | | | | |
| Astaxanthin  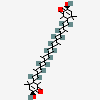 | THP-1 macrophages | 5 and 10 µM for 24 H. | ↓inflammation; ↓lipid accumulation | Downregulating the SR-A and CD36 mRNA expression | Demethylation | - | ^208-210^ |
|  | RAW264.7 macrophages | 10, 25, 50 and 100 µM for 24 H | ↑cholesterol efflux | Increasing ABCA1/G1 expression |  |  |  |
| β-Carotene  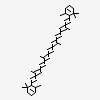 | RAW264.7 macrophages | 2 µM for 24 H | ↑cholesterol efflux | Transcriptional induction of ABCA1, ABCG1, and ApoE | Epigenetic regulator | The absolute bioavailability is about 8% after oral administration | ^211-213^ |
| Lycopene  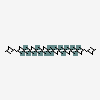 | HMDMs and THP-1 macrophages | 10 µM for 24 H | ↓lipid accumulation; ↓foam cell formation | Downregulation of the SR-A mRNA expression; increasing in the secretion of IL-10 | - | The absolute bioavailability is 1.85 +/- 0.39% after oral administration | ^214-216^ |
|  | THP-1 macrophages | 0.5, 1 and 2 µM for 24 H | ↓lipid accumulation | Involving HMG-CoA reductase inhibition, RhoA inactivation, subsequent increase in PPARγ and LXRα activation |  |  |  |
| Retinoids  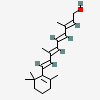 | THP-1 and RAW264.7 macrophages | 5 µM for 24 H | ↑cholesterol efflux | Inducing of ABCA1, ABCG1, and ApoE expression | Epigenetic regulator | The absolute bioavailability is about 15% after oral administration | ^217-219^ |
|  | male apoE^−/−^ mice, J774A.1 macrophages | 2 mg/kg p.o.;for 8 weeks; 0.1, 1 and 10 µM for 24 H | ↓foam cell formation; ↑cholesterol efflux | Activation of LXRα and upregulation of ABCA1 and ABCG1 expression |  |  |  |
| **Phenylpropanoids** | | | | | | | |
| Ferulic acid  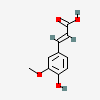 | THP-1 macrophages | 1 µM for 6 H | ↑RCT | Increasing the expression of ABCG1 and SR-BI, but not ABCA1 | - | Depends on thermal treatment of food products | ^220 221^ |
| Chlorogenic acid  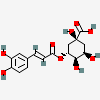 | male apoE^−/−^ mice, RAW264.7 macrophages | 200 and 400 mg/kg p.o.;for 12 weeks; 1 and 10 µM for 24 H | ↑cholesterol efflux; ↓lesion area | Increasing the transcription of PPARγ, LXRα, ABCA1, and ABCG1 | - | The absolute bioavailability is about 8% after oral administration | ^222 223^ |
| Arctigenin  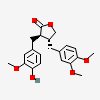 | THP-1 macrophages | 10, 50 and 100 µM for 12 H | ↑cholesterol efflux | Regulating PPARγ/LXRα/ABCA1/ABCG1 pathway | - | Low | ^224 225^ |
| Honokiol  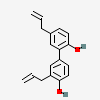 | RAW264.7 macrophages | 30 µM for 24 H | ↑cholesterol efflux | Activating the RXR/LXR heterodimer in RAW264.7 cells | - | The absolute bioavailability is 4.8 % after oral administration | ^226-228^ |
|  | U251-MG cells and THP-1 macrophages | 10 µM for 24 H | ↓lipid accumulation | Increasing ABCA1，ABCG1 and ApoE expression |  |  |  |
| Sesamin  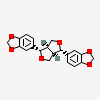 | RAW264.7 macrophages | 0.1, 1 and 10 µM for 6 H | ↑cholesterol efflux | Upregulation of the PPARγ/LXRα/ABCG1 pathway | Epigenetic regulator | The absolute bioavailability is 0.3 % after oral administration | ^229-231^ |
| **Alkaloids** | | | | | | | |
| Berberine  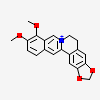 | THP-1 macrophages | 5, 10 and 20 µM for 12 H | ↑cholesterol efflux; ↓foam cell formation | Increasing LXRα/ABCA1 pathway | Epigenetic regulator | The absolute bioavailability is 0.37 ± 0.11 % after oral administration | ^232-236^ |
|  | THP-1 macrophages | 5, 10 and 20 mg/L for 2 H | ↓foam cell formation | Activating the AMPK/SIRT1/PPAR-γ pathway |  |  |  |
|  | THP-1 macrophages | 5, 10, 20, 30, 50, 70 and 100 µg/mL for 1, 2, 3, 4, 5 and 6H | ↑cholesterol efflux; ↑autophagy | Increasing ROS generation; Inducing the PI3K/AKT/mTOR pathway |  |  |  |
| Piperine  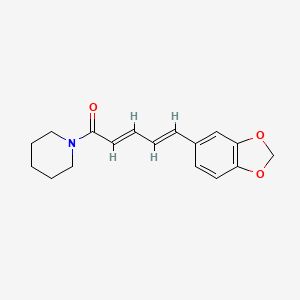 | THP-1 macrophages | 5, 10, 25, 50 and  100 µM for 24H | ↑cholesterol efflux | Upregulation of ABCA1 | Histone Modifiers | The absolute bioavailability is 25.36 % after oral administration | ^237-239^ |
| Rutaecarpine  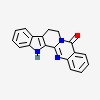 | male apoE^−/−^ mice, RAW264.7 macrophages, | 10, 20 and 40 mg/kg p.o.;for 8 weeks; 0.035, 0.35, 3.48 and 34.80 μM for 24 H | ↑RCT; ↓lesion area | Upregulated the expression of ABCA1 and SR-BI (without affecting ABCG1 and CD36) via LXRα and LXRβ | - | - | ^240^ |
| **Others** | | | | | | | |
| Astragalus polysaccharides | THP-1 macrophages | 25, 50 and 100 µg/mL for 24H | ↑cholesterol efflux; ↓inflammation | Reversing TNF-α-induced NF-κB activation | - | - | ^241^ |
| Diosgenin  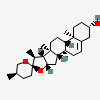 | male Albino wistar rats; THP-1 macrophages | 10, 20 and 40 mg/kg p.o.;for 8 weeks; 2, 4, 6, 8,  10, 12, 14, 16, 18 and 20 µM for 24 and 48 H | ↓foam cell formation | Inhibiting the nuclear translocation of notch intracellular domain | - | The absolute bioavailability is 9.0 ± 0.2% after oral administration | ^242-244^ |
|  | male Sprague–dawley rats; peritoneal macrophages | 25, 50 and 100 mg/kg p.o.;for 6, 7, 8, 9 and 10 weeks; 1, 2 and 4 µM for 48H | ↓foam cell formation; ↓lipid accumulation;↓inflammation | Regulating ox-LDL/LOX-1/NF-κB pathway |  |  |  |
| Panax Notoginseng Saponins | male Wistar rats | 100 mg/kg i.p.;for 9 weeks | ↓foam cell formation | Suppressing FAK phosphorylation, integrins expression and NF-κB translocation | - | Low | ^245 246^ |
| Emodin  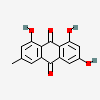 | male apoE^−/−^ mice | 901 mg/kg p.o.;for 13 weeks | ↑cholesterol efflux | Downregulating the expressions of GM-CSF and MMP-9 by activating PPAR-γ | Epigenetic regulator | The absolute bioavailability is 3.2% after oral administration | ^247-249^ |

Abbreviation: ABCA1: ATP binding cassette transporter A1, ABCG1: ATP binding cassette transporter G1, AMPK: AMP-activated protein kinase, AS: Atherosclerosis, BMDMs: Bone marrow-derived macrophages, HDL-C: High density lipoprotein cholesterol, HFD: high-fat diet, HMDMs: human peripheral blood monocyte-derived macrophages, HSC: hematopoietic stem cells, HUVECs: human umbilical vein endothelial cells, LDL-C: Low density lipoprotein cholesterol, LXRα: liver X receptor α, MAECs: Mouse aortic endothelial cells, MPMs: mouse peritoneal macrophages, PPARγ: peroxisomal proliferator-activated receptor γ, RCT: Reverse cholesterol transport, ROS: reactive oxygen species, SR-A: type A scavenger receptor, SREBPs: sterol-responsive element-binding proteins, STK11: serine/threonine kinase 11, VECs: vascular endothelial cells, VLDL-C: very low density lipoprotein cholesterol, VSMCs: vascular smooth muscle cells.
